# Asymmetric cell division is predictive of human T cell vaccine responses

**DOI:** 10.1126/sciadv.aef1608

**Published:** 2026-11-11

**Authors:** Lin Luo, Ursula K. Dueren, Ana Victoria Lechuga-Vieco, Dimitra Stamkopoulou, Liliana Tchernev, Yavuz F. Yazicioglu, Amir Hossein Kayvanjoo, Teresa Lambe, Anna Katharina Simon, Mariana Borsa

**Affiliations:** ^1^ CAMS-Oxford Institute, University of Oxford, Oxford, UK.; ^2^ Kennedy Institute of Rheumatology, Nuffield Department of Orthopaedics, Rheumatology and Musculoskeletal Sciences (NDORMS), University of Oxford, Oxford, UK.; ^3^ Department of Biomedicine, University of Basel, Basel, Switzerland.; ^4^ Max-Delbrück-Center for Molecular Medicine in the Helmholtz Association (MDC), Berlin, Germany.; ^5^ Oxford Vaccine group, Department of Paediatrics, University of Oxford, Oxford, UK.; ^6^ Pandemic Sciences Institute, University of Oxford, Oxford, UK.

## Abstract

Vaccine-induced protection relies on the generation and maintenance of stem-like memory B and T cells, a process sustained by two conserved mechanisms: asymmetric cell division (ACD) and autophagy. In T cells, ACD enables them to produce progeny with the same T cell receptor but with divergent metabolic and functional fates upon antigen encounter through the unequal inheritance of cellular cargoes. Autophagy recycles cellular components, provides metabolic substrates essential for sustaining energy demands, and is relevant for the maintenance of T cell memory. Direct evidence of how these processes synergize and affect human immunity is still lacking. Here, we show that mitotic human CD8^+^ T cells asymmetrically segregate autophagy-related organelles, including autophagosomes and lysosomes. Furthermore, we show that ACD drives divergent transcriptional and functional fates in daughter cells. Extending these findings to an influenza vaccine trial, we demonstrate that autophagic flux follows the dynamics of clonal expansion and contraction of antigen-specific T cells. Notably, individuals with enhanced ACD capacity, especially with increased LC3B asymmetry, exhibit better vaccine responses, linking early cellular segregation events to long-term immune fitness. Last, transient use of the mechanistic target of rapamycin complex 1 inhibitor rapamycin improved memory survival, which was associated with improved ACD, measured by the asymmetric inheritance of both CD8 and LC3B. Together, these findings highlight that the asymmetric inheritance of the autophagy machinery represents another layer of human adaptive immunity and offers a potential therapeutic target to enhance vaccine outcomes.

## INTRODUCTION

The magnitude and quality of vaccine-induced immunity are intimately linked to the generation of memory CD8^+^ T cells, which have self-renewing and stem-like properties essential for long-term protection (*1*). Following antigen exposure, naïve CD8^+^ T cells proliferate and differentiate into heterogeneous progeny, including short-lived terminal effector cells and long-lived memory precursors, through a tightly coordinated process that involves expansion, contraction, and survival (*2*). While substantial progress has been made to uncover the molecular programs underlying T cell differentiation during both infection and cancer, a key remaining question is the precise mechanisms guiding early fate bifurcation (*3*–*5*).

CD8^+^ T cell fate decisions are specified during the earliest stages of activation, governed by both cell-intrinsic and cell-extrinsic factors. Cell-intrinsic programs include transcriptional (*6*–*10*), metabolic (*11*–*13*), and epigenetic (*14*–*18*) mechanisms that together orchestrate lineage commitment. Cell-extrinsic cues, including T cell receptor (TCR) signal strength (*19*, *20*), cytokine availability (*21*), and tissue microenvironment (*22*, *23*) also refine the fate development trajectories. A single activated T cell is able to give rise to progeny with divergent cell fates (*24*). Asymmetric cell division (ACD) plays an important role in this scenario as it allows the emergence of daughter cells endowed with different fates. ACD is a conserved mechanism that contributes to the generation of diversity and maintenance of stem cells, which has been extensively shown in model organisms, including *Drosophila*, *Caenorhabditis elegans*, and yeast (*25*–*27*). In the mammalian immune system, murine T cells undergo ACD upon activation with antigen when the immune synapse initiates the formation of a polarity axis that enables unequal inheritance of cellular components (*24*, *28*–*33*). Daughter cells proximal to the antigen-presenting cell (APC) acquire more effector-associated cargo, whereas distal daughters are more likely to exhibit a memory fate (*28*). Although recent work has extended the relevance of ACD to in vitro–generated human antitumor CAR-T cells (*34*), key questions remain regarding its physiological role in regulating human T cell fate. Furthermore, the asymmetrically inherited fate determinants in this context are yet to be identified, and the functional significance of ACD for human immune responses is critical for future studies.

Among potential candidates, the cellular degradation machinery, including proteasomes, lysosomes, and the autophagy/lysosome system (macroautophagy, hereafter autophagy) has emerged as a regulator of fate specification in stem cells and immune cells (*31*, *35*, *36*). In particular, autophagy maintains T cell homeostasis by preserving mitochondrial health (*37*, *38*), recycling intracellular components, and supporting cell fate of both effector and memory subsets (*39*–*44*). Autophagy deficiency impairs memory formation in mice and compromises T cell immune responses in humans (*39*, *45*), raising the question of how autophagy affects the early fate commitment in human T cells.

Here, we focused on first mitotic human T cells in healthy donors including those who had received viral-vectored vaccines and identified asymmetric inheritance of cell cargoes, including the autophagic machinery, as an important mechanism in maintaining human CD8^+^ T cell diversity. Using confocal microscopy, we show that mitotic human CD8^+^ T cells asymmetrically segregate autophagy-related organelles, including autophagosomes and lysosomes. We also provide evidence that, similar to what has been shown in murine T cells, ACD drives divergent transcriptional and functional fates in human T cells. Benefiting from an investigational influenza viral-vectored vaccine trial, we extended these findings to the analysis of T cell features in humans by characterizing ex vivo T cell phenotype and function across baseline and postvaccination time points. We used a flow cytometry–based autophagic flux assay and metabolic readouts and showed that autophagic flux dynamically correlates with the clonal expansion of antigen-specific T cells during an influenza vaccine response. Notably, CD8^+^ T cells from time points when T cell quiescence is expected underwent more ACD at first division following activation. Individuals with greater ACD capacity exhibited higher vaccine responses as measured by T cell enzyme-linked immunospot (ELISpot), directly linking this mechanism to long-term immune fitness during human immune responses. Furthermore, transient inhibition of mechanistic target of rapamycin complex 1 (mTORC1) by rapamycin enhanced ACD capacity, leading to improved memory cell survival.

## RESULTS

### Human CD8^+^ T cells acquire divergent phenotypical and functional profiles following ACD

To investigate whether ACD plays a role in human CD8^+^ T cells, we isolated CD8^+^ T cells from healthy donors’ peripheral blood mononuclear cells (PBMCs) and analyzed their first division after in vitro activation with anti-CD3 and anti-CD28. The first cell division was typically observed 48 to 52 hours after activation (Fig. 1A and fig. S1A), which we used as the window for assessing mitosis. Stable T cell engagement with APCs relies on the interaction between leukocyte function-associated antigen-1 (LFA-1) and intercellular adhesion molecule 1 (ICAM-1) and is essential for ACD to occur (*28*). To mimic this process in vitro, we used Fc–ICAM-1 during our plate-bound activation, in combination with anti-CD3 and anti-CD28, to observe T cell asymmetry as previously described (*32*, *33*, *46*). We then evaluated the inheritance of CD8, a reference molecule known to be segregated asymmetrically upon ACD in mitotic cells, identified by the presence of a tubulin bridge and condensed chromosome morphology as previously described (*32*, *33*). As expected, we observed CD8 asymmetry in mitotic cells in stimulation conditions in the presence of Fc–ICAM-1, as opposed to controls lacking Fc–ICAM-1 (Fig. 1, B and C). DAPI (4′,6-diamidino-2-phenylindole) intensity was confirmed to remain symmetric across conditions, supporting that CD8 asymmetry is induced specifically in the presence of Fc–ICAM-1 (Fig. 1C). Following the detection of ACD, we assessed whether this asymmetry influenced T cell fates in an established in vitro model of human memory T cell differentiation (*47*). After activation for 48 to 52 hours, T cells were cultured in vitro for 7 days in medium containing interleukin-15 (IL-15) to benefit memory differentiation and maintenance. We observed a higher proportion of CD62L^+^CD27^+^ cells, which are phenotypic markers for memory T cells, in progeny derived from cells that were previously stimulated in the presence of Fc–ICAM-1, when compared to those stimulated in the absence of Fc–ICAM-1 (Fig. 1D), suggesting that, as previously seen in murine T cells (*28*, *32*), this integrin sustains human T cells to undergo ACD and supports the generation of memory progeny. We also confirmed the phenotypes by staining T cell factor 1 (TCF-1) and Forkhead box protein O1 (FOXO1) (fig. S1B). Consistently, we observed higher frequencies of CD62L^+^CCR7^+^, CD62L^+^TCF-1^+^, and CD62L^+^FOXO1^+^ populations in progeny of Fc-ICAM-1–activated cells (fig. S1, C to E), corroborating the contribution of ACD to the rise of cells with memory-like phenotypes.

**Fig. 1.**
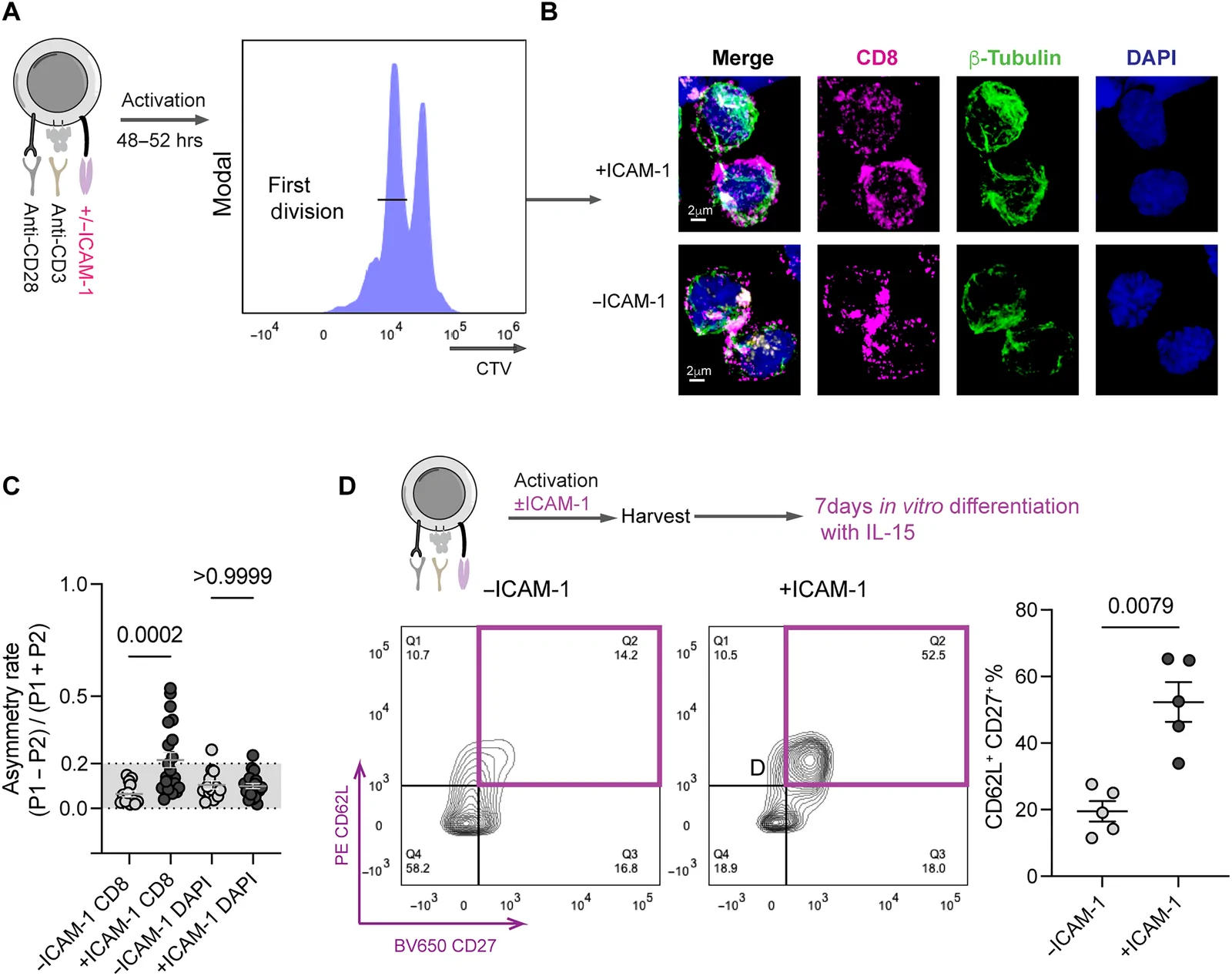
Human CD8^+^ T cells undergo ACD in the presence of Fc–ICAM-1. (**A**) CellTrace Violet (CTV)–labeled CD8^+^ T cells were activated with anti-CD3 and anti-CD28 in the presence or not of Fc–ICAM-1 for 48 to 52 hours (hrs) to obtain first-division daughter cells, identified by the first CTV dilution peak. (**B**) Representative confocal images of mitotic human CD8^+^ T cells after 48 to 52 hours of activation, at their first division. Asymmetry of CD8 and DAPI was measured within the mitotic pairs. (**C**) Quantification of CD8 and DAPI asymmetry from B using the ratio (P1 − P2) / (P1 + P2). (−Fc–ICAM-1: *n* = 13; +Fc–ICAM-1: *n* = 21). Any value above 0.2 was classified as ACD. (**D**) Cells were activated with anti-CD3 and anti-CD28 in the presence or not of Fc–ICAM-1 for 48 to 52 hours and cultured for 7 days in the presence of IL-15 (10 ng/ml). Representative flow cytometry plots (left) and quantified frequencies of CD62L^+^CD27^+^ cells. Each data point represents one pair of mitotic cells analyzed (C) or one healthy donor (D). Data are presented as means ± SEM. Statistical analysis was performed using Kruskal-Wallis (C) and Mann-Whitney test (D). Representative data of one of two experiments.

To further explore functional differences between first-daughter cells following ACD, we sorted them into CD8 high (CD8 hi) and CD8 low (CD8 lo) populations as previously described in murine T cells (*32*) and cultured them in conditions with limited availability of IL-15 (1 ng/ml) (Fig. 2A). We observed that CD8 lo progeny showed better survival than CD8 hi progeny as defined by cell viability by live/dead staining (Fig. 2, B and C). In contrast, when we tracked the proliferation profile of CD8 hi and CD8 lo daughter cells 2 or 6 days after sorting, CD8 hi cells exhibited a faster proliferation rate than CD8 lo cells (Fig. 2D), with a significantly higher proportion undergoing multiple divisions (>1 division) within 2 days (Fig. 2E). These data suggest CD8 hi cells resemble more effector cells, while CD8 lo cells resemble memory T cells following ACD in this in vitro differentiation model. To further explore distinct features known to characterize effector and memory first daughter cells, we measured protein synthesis using a click chemistry–based assay. Here, the fluorescent puromycin analog *O*-propargyl-puromycin (OPP) is incorporated into nascent proteins, which can be measured by flow cytometry (fig. S1F) (*48*). We observed that CD8 hi cells exhibited higher translation activity following the first mitosis of human CD8^+^ T cells when compared to their CD8 lo counterparts (Fig. 2F and fig. S1G), consistent with the high proliferation demands of effector T cells.

**Fig. 2.**
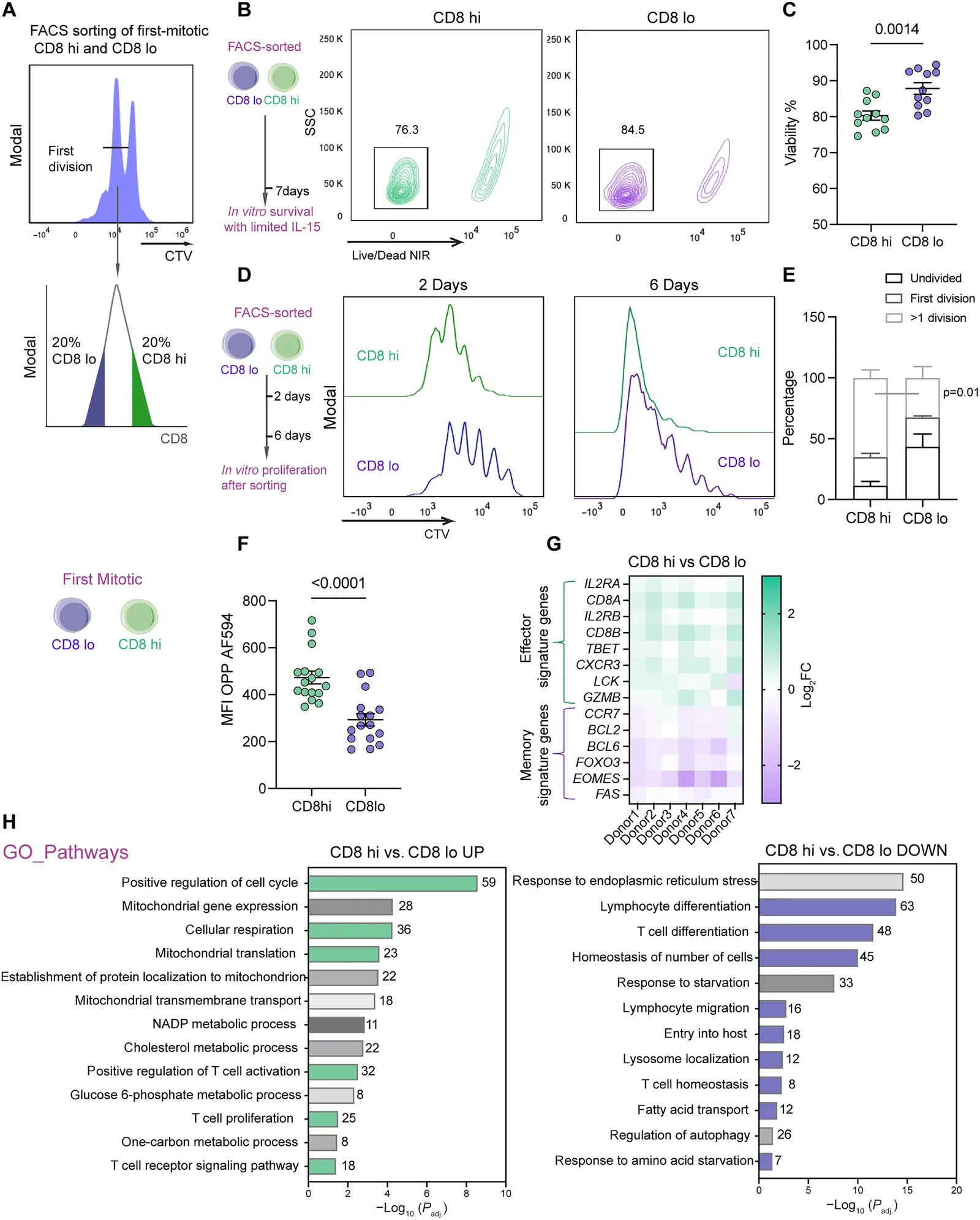
Human CD8 T^+^ cells exhibit different phenotype and function following ACD. (**A**) Experimental layout: CTV-labeled cells were activated for 48 to 52 hours. Cells were harvested and stained with anti-CD8 antibody and a cell viability dye. First-daughter cells were identified as the first peak of CTV dilution (top). CD8 hi and CD8 lo cells were sorted as populations with the 20% highest or lowest CD8 expression (CD8 hi and CD8 lo) (top). (**B**) CD8 hi and CD8 lo cells were cultured in vitro for 7 days in medium containing IL-15 (1 ng/ml). Representative flow cytometry plots showing cell viability of sorted CD8 hi and CD8 lo progenies after 7 days in medium containing IL-15 (1 ng/ml). (**C**) Quantification of survival rates in CD8 hi and CD8 lo cells from (B), *n* = 11. (**D**) Representative flow cytometry histograms showing proliferation profile of sorted CD8 hi and CD8 lo progenies [from (A)]. Cells were cultured in vitro in medium containing IL-2 (50 U/ml) for 2 or 6 days. (**E**) Quantification of frequencies of cells [from (D)] that did not divide, divided once, or underwent more than one division after being cultured for 2 days in vitro, *n* = 3. (**F**) Quantification of OPP incorporation in CD8 hi and CD8 lo first daughter cells. (**G**) Heatmap showing fold-change expression ratios of effector and memory-associated genes among healthy donors. Heatmap is plotted by Log_2_FC. (**H**) Gene Ontology (GO) pathway enrichment analysis of DEGs in CD8 hi versus CD8 lo cells revealed enrichment of biological pathways associated with either effector-like (green) or memory-like (purple) T cell fates. Each data point represents one healthy donor. Data are presented as means ± SEM. Statistical analysis was performed using an unpaired *t* test with Welch’s correction in (C), Two-way analysis of variance (ANOVA) with Bonferroni’s correction in (E), and Mann-Whitney in (F). Data are pooled from two to three experiments in (C) and representative of one experiment of three in (E).

Last, to explore the molecular differences between CD8 hi and lo cells, we performed RNA sequencing on CD8 hi and CD8 lo first daughter cells from seven healthy donors. Principal component analysis revealed distinct clustering between CD8 hi and CD8 lo groups (fig. S1H). Differentially expressed genes (DEGs) analysis identified 2298 DEGs. Among these genes, *IL2RA, CD8A, IL2RB, CD8B, TBX21, CXCR3, LCK,* and *GZMB* were up-regulated in CD8 hi cells matching their effector-like identity (*8*, *32*). In contrast, *CCR7, BCL2, BCL6, FOXO3, EOMES,* and *FAS *were up-regulated in CD8 lo cells, matching their memory-like identity (Fig. 2G) (*8*, *32*). Gene Ontology enrichment analysis of up-regulated genes in CD8 hi cells revealed significant associations with biological processes related to positive regulation of cell cycle, T cell proliferation and activation, TCR signaling, mitochondrial gene expression, cellular respiration, mitochondrial translation, nicotinamide adenine dinucleotide phosphate metabolism, and cholesterol metabolism (Fig. 2H, left, highlighted in green), matching their effector-like cell fate. Conversely, genes up-regulated in CD8 lo cells were enriched in pathways related to endoplasmic reticulum (ER) stress responses, T cell differentiation, homeostasis, fatty acid transport, and autophagy regulation (Fig. 2H, right, highlighted in purple), matching their memory-like cell fate. Collectively, our findings demonstrate that ACD in human CD8^+^ T cells results in phenotypically and functionally distinct daughter cells. CD8 hi cells exhibit features of effector-like cells with enhanced proliferation and translation activity, while CD8 lo cells show a memory-like transcriptome and enhanced survival capacity, highlighting the role of ACD in directing early divergent cell fates in human CD8^+^ T cells.

### The autophagic machinery is asymmetrically inherited in human CD8^+^ T cells

Cellular degradation mechanisms are essential for maintaining cell fate and metabolic fitness (*49*, *50*). In the context of murine ACD, it has been shown that CD8 lo cells inherit more proteasomes, which by degrading T-bet, a key transcription factor for differentiation of effector cells, bias the fate of these cells toward becoming memory-like (*31*). Thus, we hypothesized that the autophagic machinery, a key system that selectively degrades intracellular cargo and recycles nutrients to regulate cell fate and function in the immune system (*51*), may also play a role in human CD8^+^ T cell fate divergence. To investigate this, we first assessed the distribution of autophagosomes and lysosomes during the first division of activated CD8^+^ T cells by immunofluorescence staining of LC3B (autophagosome marker) and LAMP1 (lysosome marker), followed by confocal microscopy analysis. We observed that both LAMP1 and LC3B were asymmetrically inherited (Fig. 3, A and B). Furthermore, the degree of LC3B and LAMP1 asymmetry moderately correlated (*r* = 0.38, 0.25, respectively) with CD8 asymmetry within dividing cell pairs (fig. S2, A and B). We also confirmed that this asymmetry was not affected by transient autophagy inhibition by bafilomycin A1 (BafA) (fig. S2C). We next evaluated whether this asymmetric distribution was linked to the functional and phenotypic divergence observed in CD8 hi and CD8 lo cells. By using a flow cytometry–based assay (fig. S2D), we measured autophagic flux in these two populations during in vitro differentiation. As autophagy has been shown to be important for memory CD8^+^ T cells (*39*, *40*, *44*, *45*), we measured autophagy in CD8 hi and CD8 lo progenies 3 and 7 days after first division. CD8 lo cell progeny exceeded the autophagic activity of their CD8 hi counterparts 3 days following cell division (Fig. 3C and fig. S2E). By day 7 during in vitro differentiation, both CD8 hi and CD8 lo progenies exhibited reduced autophagy levels, measured by autophagic flux, in comparison to their day 3 counterparts. Thus, although CD8 lo cells exhibit higher autophagic flux at day 3, at day 7, both CD8 hi and CD8 lo cells showed comparable autophagy levels (Fig. 3C and fig. S2F). This can be explained by an enrichment of memory-like cells in both conditions, although in lower viability when emerging from CD8 hi cells. We observed higher expression of autophagy-related genes in CD8 lo cells when the transcriptional profile of first daughter cells was evaluated (fig. S2G), indicating that ACD promotes unequal inheritance of transcripts related to autophagic machinery, which perhaps support the increased autophagy rates observed in their progeny days later. To understand the role of autophagy in regulating early daughter cell fate, we sorted the first daughter CD8 hi and CD8 lo cells and cultured them in the presence or absence of chloroquine (CQ). Following activation and sorting of first-division CD8 hi and CD8 lo cells, cells were rested for 24 hours before being treated or not with CQ for 3 days (fig. S3A). CQ treatment resulted in equally reduced viability in both populations when compared to the untreated groups (fig. S3B). Further phenotypic analysis of the surviving CD8 hi and CD8 lo cells revealed significant reduction in the frequency of memory-like cells (CD62L^+^CD27^+^) following CQ treatment (fig. S3, C and D) but not the frequency of effector-like (CD25^+^CD44^+^) cells (fig. S3, E and F), suggesting CQ preferentially affects the memory-like population.

**Fig. 3.**
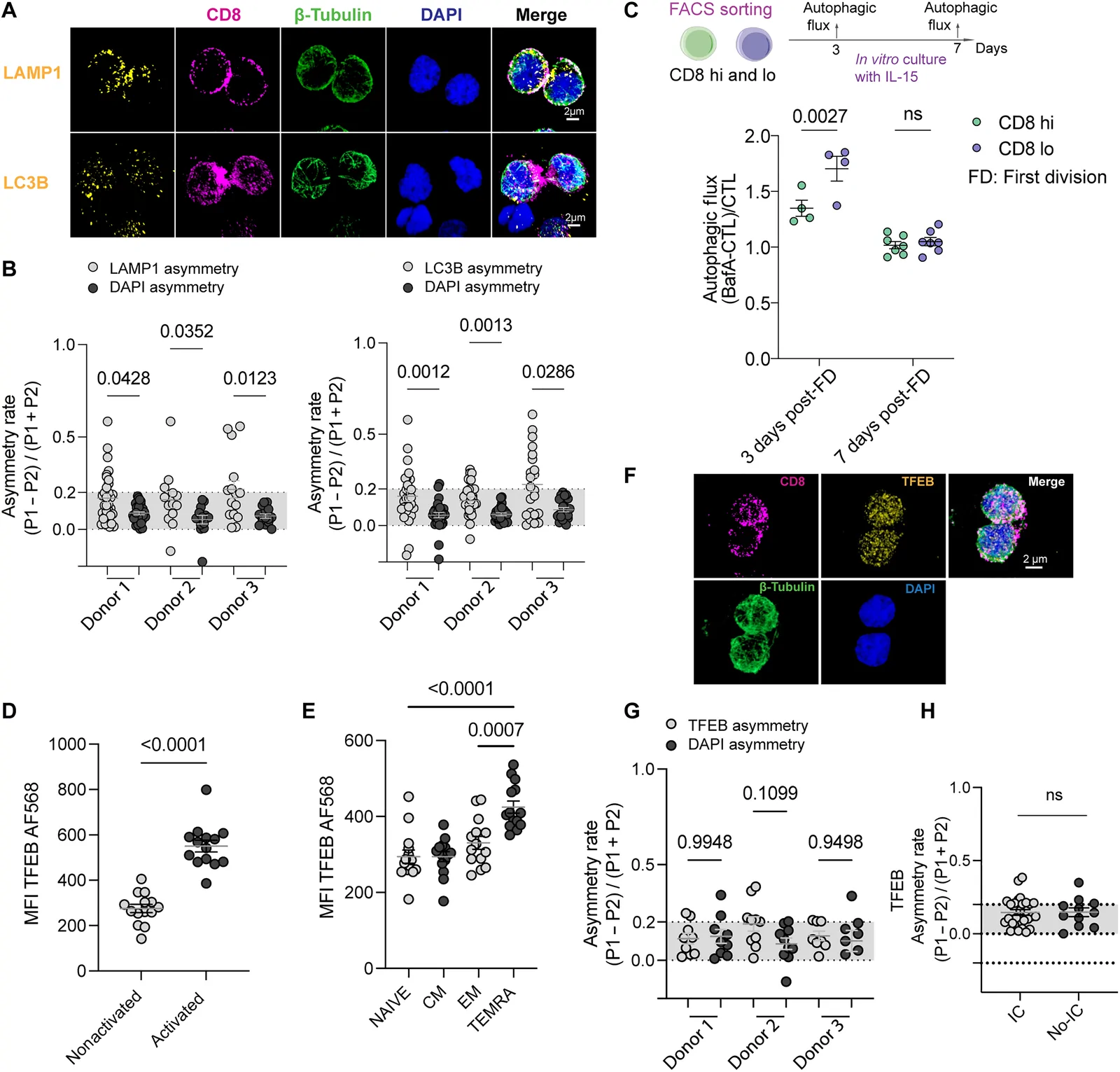
The autophagic machinery is asymmetrically inherited in human CD8^+^ T cells. (**A**) Representative confocal images of mitotic CD8^+^ T cells 48 hours postactivation. (**B**) Quantification of LAMP1 (*n*D1 = 32, *n*D2 = 13, *n*D3 = 15) and LC3B (*n*D1 = 28, *n*D2 = 28, *n*D3 = 22) asymmetry, compared to DAPI (symmetric control) in total CD8^+^ T cells derived from three healthy donors. Any value above 0.2 was classified as ACD. (**C**) Autophagic flux was measured by calculating the following ratio of LC3-II MFI: (BafA-CTL)/CTL. This was done at two different time points: 3 and 7 days postsorting on first mitotic CD8 hi and CD8 lo cells (top). Time course of autophagic flux of sorted CD8 hi and CD8 lo cells originated from conditions promoting ACD (+ICAM) (bottom). (**D**) TFEB expression levels in unstimulated or activated total CD8^+^ T cells, *n* = 14. (**E**) TFEB expression levels in T cell subsets, *n* = 14. (**F**) Representative confocal images of mitotic CD8^+^ T cells after 48 to 52 hours of activation. TFEB distribution was measured in the mitotic pairs. (**G**) Quantification of TFEB asymmetry compared to DAPI (symmetric control) in three healthy donors (*n*D1 = 9, *n*D2 = 10, *n*D3 = 8). (**H**) Comparison of TFEB asymmetry following CD8^+^ T cell activation with (*n* = 27) or without (No-IC, *n* = 11) Fc–ICAM-1. Each data point represents one healthy donor [(C), (D), and (E)] or one pair of mitosis analyzed [(B), (G), and (H)]. Data points with the same shape represent samples from the same donor. Data are presented as means ± SEM. Statistical analysis was performed using Kruskal-Wallis with multiple-comparisons test [(B) and (G)], two-way ANOVA with Bonferroni’s correction (C), unpaired Student’s *t* test (D), and Kruskal-Wallis in [(E) and (H)]. Exact *P* values are depicted in the figure. Data are pooled from two to three experiments [(D) and (E)] or representative of one of two experiments [(C) and (H)].

We then hypothesized that the differences in expression of autophagy-related genes might be promoted by an upstream regulator, the transcription factor EB (TFEB). TFEB orchestrates lysosomal biogenesis and autophagy gene expression (*52*). We observed a clear up-regulation of TFEB expression during in vitro CD8^+^ T cell activation (Fig. 3D) and ex vivo in terminally differentiated effector memory cells reexpressing CD45RA (TEMRA) CD8^+^ T cell subsets compared to their naïve or memory counterparts (Fig. 3E and fig. S4A), which might contribute to the up-regulated autophagic flux in this subset previously reported by others (*53*). However, we could not observe asymmetric TFEB distribution during CD8^+^ T cell mitosis (Fig. 3, F to H). In summary, components of the autophagic machinery are asymmetrically inherited, and it is these, not asymmetric inheritance of a transcription factor nor transcriptional regulation of autophagy, that may predict divergent fates between CD8 hi versus CD8 lo cell progenies.

### Autophagic machinery is induced during human T cell memory recall responses

To extend our findings to a physiological human immune response, we analyzed samples from a previously described influenza vaccine trial with a candidate vaccine in which participants received one dose of ChAdOx1 H3N2 nucleoprotein (NP) + M1 on day 0 (D0) with or without the booster dose of MVA H3N2 NP + M1 vaccine on day 56 (D56) (Fig. 4A) (*54*). The trial is registered at www.clinicaltrials.gov (Identifier: NCT01818362). Antigen-specific CD8^+^ T cells were identified by influenza tetramers, which are fluorophore-conjugated streptavidin molecules bound to biotinylated major histocompatibility complex I molecules loaded with influenza peptides [human leukocyte antigen (HLA)*A1:01: CTELKLSDY bound to NP [44-52]; HLA*A2:01: GILGFVFTL bound to M1 [58-66], provided by T. Dong, University of Oxford] and analyzed by spectral flow cytometry (fig. S4B). Comparison between tetramer-positive (Tet^+^) and tetramer-negative (Tet^−^) populations revealed that autophagic flux was markedly elevated in Tet^+^ cells during the vaccination course at days 0, 7, 56, 63, and 182, in comparison to Tet^−^ cells. This difference was most pronounced at day 63 (D63), corresponding to the memory recall response following the second vaccine dose (Fig. 4B). These findings are consistent with previous results from our group analyzing samples from a respiratory syncytial virus (RSV) vaccine trial (*45*). To further understand which cell population contributes to the up-regulated autophagic flux upon vaccination, we analyzed the phenotype of human CD8^+^ T cells within Tet^+^ cells, which were subdivided into CD45RO^+^ and CD45RA^+^ populations, representing predominantly memory-like and effector-like (effector and TEMRA cells) cells, respectively. We found that approximately 70% of Tet^+^ cells exhibited a memory-like phenotype (CD45RO^+^) as expected, whereas CD45RA^+^ cells accounted for less than 20% of this population (fig. S4C), suggesting that the Tet^+^ cells are mainly memory-like cells and contribute to elevated autophagy (fig. S4D). We observed that TFEB expression was also higher in Tet^+^ cells at time points following antigen encounter (Fig. 4C). Together, these data suggest that the autophagic machinery is specifically up-regulated in antigen-specific CD8^+^ T cells following vaccination. This could support the enhanced metabolic demands of these antigen-experienced cells, which predominantly rely on glycolysis for effector cells and/or fatty acid oxidation (FAO) for memory cells (*55*, *56*). Thus, we assessed mitochondrial and metabolic features in the same trial samples. We first evaluated glucose uptake capacity by measuring surface GLUT1 expression, a key glucose transporter in T cells. Similarly to autophagy activity, GLUT1 expression significantly increased in Tet^+^ cells, particularly during the effector (D7) and memory recall (D63) phases of the vaccine response (Fig. 4D). Given the well-established role of autophagy in maintaining mitochondrial quality and supporting metabolic homeostasis (*57*, *58*), we examined the expression of TOM20, a mitochondrial outer membrane marker that can be used to measure mitochondrial content per cell, but we did not observe any differences across time points in neither Tet^+^ nor Tet^−^ populations (Fig. 4E). We further examined the expression of carnitine palmitoyltransferase 1a (CPT1A), a rate-limiting enzyme important for mitochondrial fatty acid import and a hallmark of FAO, and observed increased levels in Tet^+^ cells upon recall responses (Fig. 4F), supporting existing evidence on the reliance of memory cells on FAO (*55*, *59*).

**Fig. 4.**
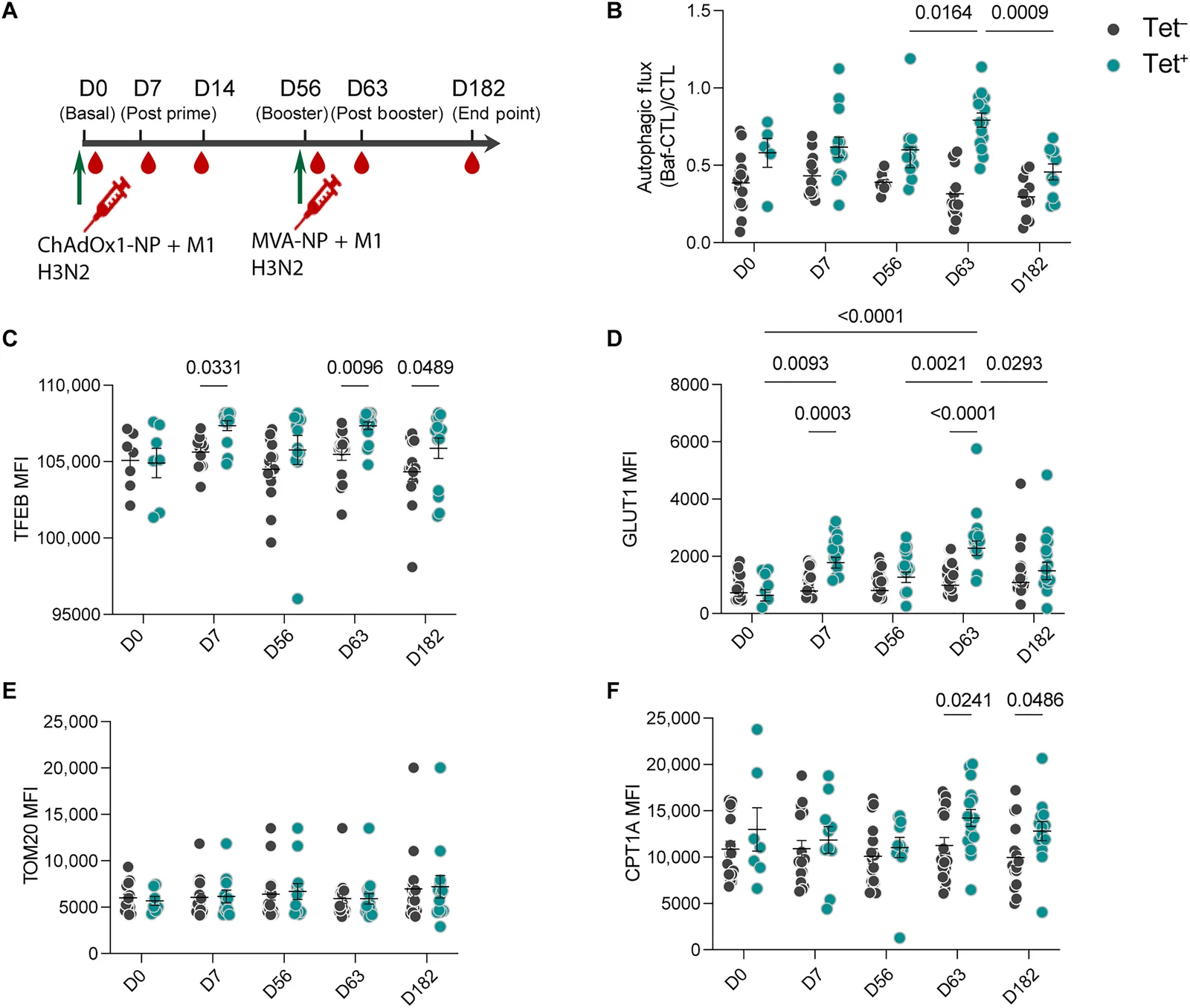
Autophagic machinery is induced during human T cell memory recall responses. (**A**) Vaccination regimen of influenza vaccine trial (FLU005) with two doses of M1 + NP H3N2 vaccine using ChAdOx1 or MVA platforms. (**B**) Autophagic flux was measured and calculated in Tet^+^ and Tet^−^ cells during vaccination time course—basal (D0), postprime (D7, D14), before booster (D56), postbooster (D63), and at end point (D182). (**C** to **F**) Intensity of TFEB, GLUT1, TOM20, and CPT1A expression in Tet^+^ and Tet^−^ CD8^+^ T cells during the course of immune responses in vaccinees. Each data point represents one participant from the FLU005 trial. Data are presented as means ± SEM. Statistical analysis was performed using two-way ANOVA with Tukey’s correction [(B) to (F)]. Exact *P* values are depicted in the figure, *n* = 20 [(B) to (F)].

### ACD correlates with human vaccine responses

ACD is a mechanism that contributes to T cell diversity and, in murine models, is more pronounced in multipotent cell types (naïve, central memory, and virtual memory) and less frequent in terminally differentiated cells that are not able to self-renew, such as effector or exhausted cells (*32*, *33*). To explore whether this principle applies to human T cells, we examined ACD dynamics in total CD8^+^ T cells during the course of influenza vaccination using samples from donors that received one dose of ChAdOx1 M1 + NP H3N2 influenza vaccine followed or not by a booster dose of MVA M1 + NP H3N2 as described before (FLU005) (Fig. 5A) (*54*). On the basis of mouse studies, quiescent T cell subsets are prone to undergo ACD while effector/exhausted T cells are prone to undergo symmetric cell division, respectively (*32*). We isolated and activated CD8^+^ T cells from either quiescent (D0, D182) or effector phase (D14) during immune responses using anti-CD3, anti-CD28, and Fc-ICAM-1 as described above and evaluated their asymmetry rates (Fig. 5B). Quiescent samples were analyzed at baseline (D0) and memory phase (D182) and peak effector phase was assessed 2 weeks following vaccination (D14). ACD was quantified at each time point by measuring CD8 inheritance in mitotic pairs (Fig. 5C). We did not observe significant changes in CD8 asymmetry across different time points during vaccine-elicited immune responses. We initially hypothesized that asymmetry rates would be lower during the expansion of effector T cells as this has been previously reported in murine CD8^+^ T cells (*32*). Frequencies of T cell subsets during vaccination remained largely unchanged across different time points and donors in this cohort (fig. S5A). Concerning antigen-specific cells, we observed the expected accumulation of Tet^+^ cells at D14, which confirms T cell expansion during the effector phase of an immune response (fig. S5B). However, as antigen-specific cells constitute a small fraction of the total CD8^+^ T cell population, ACD rates were not affected by the relatively small fluctuations in their frequencies. As we observed the most significant CD8 asymmetry at D0 (before vaccination), we nevertheless chose this time point as the baseline to assess ACD potential in CD8^+^ T cells of different donors from this cohort.

**Fig. 5.**
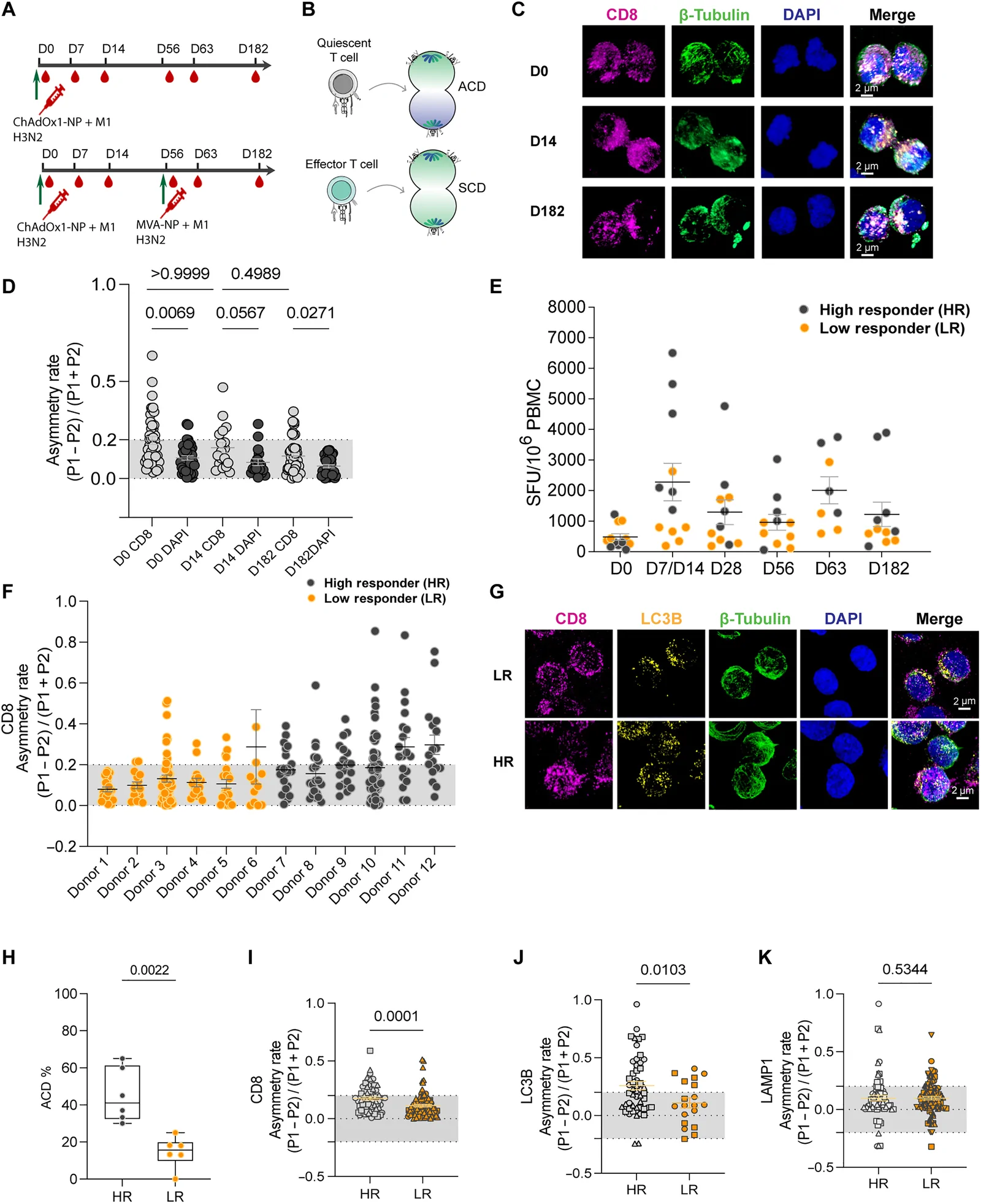
Asymmetric inheritance of autophagic machinery is important for human vaccine responses. (**A**) Vaccination regimen of influenza vaccine trial (FLU005). Two doses of M1 + NP H3N2 vaccine were delivered to individuals with ChAdOx1 and MVA platforms, respectively. (**B**) Graphical schematic of the asymmetry potential of quiescent T cells and effector T cells (hypothesis based on previous studies in murine T cells). (**C**) Representative confocal images of mitotic CD8^+^ T cells isolated from PBMCs of FLU005 vaccines at different time points: D0, D14, and D182. Cells were activated with anti-CD3, anti-CD28, and Fc-ICAM-1 and imaged 48 to 52 hours later. (**D**) CD8 asymmetry rates exhibited by first-division daughter cells were compared to their internal symmetry control, DAPI, *n* = 42 (D0), 18 (D14), 39 (D182). (**E**) T cell ELISpot was performed, and T cell responses were calculated as fold-change of SFU at D7 or D14 versus D0. (**F**) CD8 asymmetry was quantified in total CD8^+^ T cells isolated from PBMCs across HRs and LRs from the FLU005 trial. Any value above 0.2 was classified as ACD. (**G**) Representative confocal images of mitotic CD8^+^ T cells 48 to 52 hours postactivation. CD8 and LC3B asymmetries were calculated in HR (high responders) and LR (low responders). (**H**) Frequency of mitoses with asymmetric CD8 inheritance (ACD%) was calculated as the ratio between the number of asymmetry pairs/number of total mitotic pairs across donors. ACD% was calculated and compared between HR and LR. (**I**) CD8 asymmetry was calculated and compared between HR (*n*D1 = 21, *n*D2 = 19, *n*D3 = 18) and LR (*n*D1 = 15, *n*D2 = 21, *n*D3 = 49). (**J**) LC3B asymmetry was calculated and compared between HR (*n*D1 = 17, *n*D2 = 19, *n*D3 = 14) and LR (*n*D1 = 10, *n*D2 = 9). (**K**) LAMP1 asymmetry was calculated and compared between HR (*n*D1 = 19, nD2 = 31, *n*D3 = 20) and LR (*n*D1 = 15, *n*D2 = 14, *n*D3 = 22, *n*D4 = 19, *n*D5 = 21). Each data point represents one healthy donor [(E) and (H)] or one pair of mitosis analyzed [(D), (F), (I), (J), and (K)]. Data points with the same shape represent samples from the same donor. Data are presented as means ± SEM [(D), (F), (I), (J), and (K)] or min to max (H). Statistical analysis was performed using Kruskal-Wallis test (D) or Mann-Whitney test [(H), (I), (J), and (K)]. Exact *P* values are depicted in the figure. Data are pooled from at least three experiments in [(F), (G), (H), (I), (J), and (K)].

CD8^+^ T cells are heterogeneous in humans, and differences in frequencies of specific subsets across individuals could affect the capacity of total CD8^+^ T cell populations to undergo ACD. As subset-specific analyses of ACD were not possible because of cell number limitations in samples from the clinical trial, we compared the ACD levels between total and naïve CD8^+^ T cells in healthy donors and did not observe significant differences in asymmetry levels between them (fig. S5C). Thus, for samples from vaccinees, we have used total CD8^+^ T cells for mitotic analysis and ACD measurements. Next, we sought to investigate the correlation of ACD rates and vaccine responses. Vaccine-induced T cell responses were assessed using interferon-γ (IFN-γ) ELISpot assays, a standard readout for viral vector–based vaccines. Vaccine responsiveness was quantified as the fold change in IFN-γ–secreting T cells after vaccination compared with baseline (D7 or D14 versus D0) (Fig. 5E). According to the vaccine responsiveness, a threshold of high (HR) or low responders (LR) was set on the basis of the median fold change (fig. S5D). We then measured frequencies of ACD within mitotic cells in 12 samples at baseline (D0) (Fig. 5F) and observed a significant correlation to individual’s vaccine responses (fig. S5E). We found that the individuals with higher vaccine responsiveness (the HR group) exhibited significantly higher ACD rates (measured by CD8 segregation) in comparison to the ones with lower vaccine responsiveness (LR group) (Fig. 5, G and H). Although both young and old participants were recruited in this trial, we did not observe a significant age-related decline in ACD level and T cell response (fig. S5, F and G). This is perhaps because the older participants in this group were younger than 65 years old, an age commonly acknowledged as the threshold for immunosenescense (*60*). At the cellular level, both CD8 and LC3B, but not LAMP1 asymmetry, were significantly higher in the HR group compared to the LR group (Fig. 5, I to K). However, autophagic flux levels were similar between LR and HR individuals (fig. S5H). These findings suggest that the capacity to undergo ACD, including the asymmetric inheritance of autophagic machinery—rather than absolute autophagic levels—may serve as an early indicator of effective memory T cell generation and antigen-specific T cell response following vaccination.

### Enhanced ACD boosts T cell memory survival and maintenance

Given the observed positive correlation between ACD and vaccine responsiveness, we aimed to determine whether modulating ACD could enhance the generation of memory progeny and thereby benefit T cell immunity. Transient treatment with rapamycin has been found to improve ACD rates in different cell types, from yeast to murine and human CD8^+^ T cells, by promoting ER diffusion barrier formation (*32*, *61*, *62*). Rapamycin is also a promising antiaging drug given its beneficial effects on extending health span and life span (*63*, *64*), inducing autophagy in some cell types (*65*, *66*), limiting protein translation and amino acid transport (*67*), and DNA damage (*68*). Rapamycin treatment has also been shown to significantly improve vaccine response in humans (*69*–*71*). In our research, we treated human CD8^+^ T cells with low-dose rapamycin (20 nM) 12 hours after activation and harvested cells at the time of first mitosis to assess ACD (Fig. 6A). We firstly observed increased frequencies of asymmetric inheritance of CD8 (Fig. 6A), which is consistent with our previous studies (*32*). We also observed higher rates of LC3B asymmetry in mitotic cells following rapamycin treatment (Fig. 6A), suggesting that transient mTOR inhibition may also promote asymmetric inheritance of the autophagic machinery. To evaluate the functional consequences of this modulation, and whether it would benefit the generation of cells endowed with memory potential, we assessed T cell survival as previously described (*32*). After activation with or without rapamycin treatment, cells were harvested and cultured in conditions with limited IL-15 availability for 5 days. Cell viability was then measured as a proxy for memory potential (Fig. 6B). Even a transient rapamycin treatment resulted in significantly enhanced T cell survival (Fig. 6B), accompanied by a higher proportion of CCR7^+^CD62L^+^ cells, consistent with a central memory-like phenotype (Fig. 6B). The improved survival and increased frequency of CCR7^+^CD62L^+^ cells were not due to enhanced proliferation (fig. S6, A to C). This survival advantage in progeny of rapamycin-treated cells was not accompanied by a sustained increase in autophagic flux in total CD8^+^ T cells or within the CCR7^+^CD62L^+^ population (fig. S6, D and E), aligning with our earlier observation that asymmetric inheritance of autophagy machinery, rather than overall autophagy level, may be contributing to memory T cell maintenance. To test whether the benefits of rapamycin were specifically mediated through ACD, we administered the treatment after the first division was completed (Fig. 6C). Under this condition, rapamycin failed to improve either T cell survival or the frequency of memory-like cells, suggesting that the timing of pharmacological modulation is critical and that the first mitotic division is a decisive window for programming memory progeny through ACD. To further test whether the effects of rapamycin were exerted through autophagy, CD8^+^ T cells were activated with anti-CD3, anti-CD28, and Fc–ICAM-1 and drugs (control, rapamycin, and rapamycin plus CQ) were supplemented 12 to 16 hours postactivation and maintained for a total period of 32 to 36 hours. The cells were then cultured in vitro in IL-15 (1 ng/ml) survival media for 5 days (fig. S6F), after which viability was assessed. We found that transient rapamycin treatment increased T cell viability after 5 days (fig. S6G). However, we did not observe a significant difference in survival between the rapamycin (RP) and rapamycin + CQ (RP + CQ) groups, indicating that CQ-mediated inhibition of autophagy does not abrogate the prosurvival effect of rapamycin (fig. S6G). We also attempted to increase CQ concentration (up to 10 mM) to achieve stronger autophagy inhibition. However, higher CQ concentrations resulted in substantial T cell death, thereby limiting its utility in long-term survival assays (fig. S6H).

**Fig. 6.**
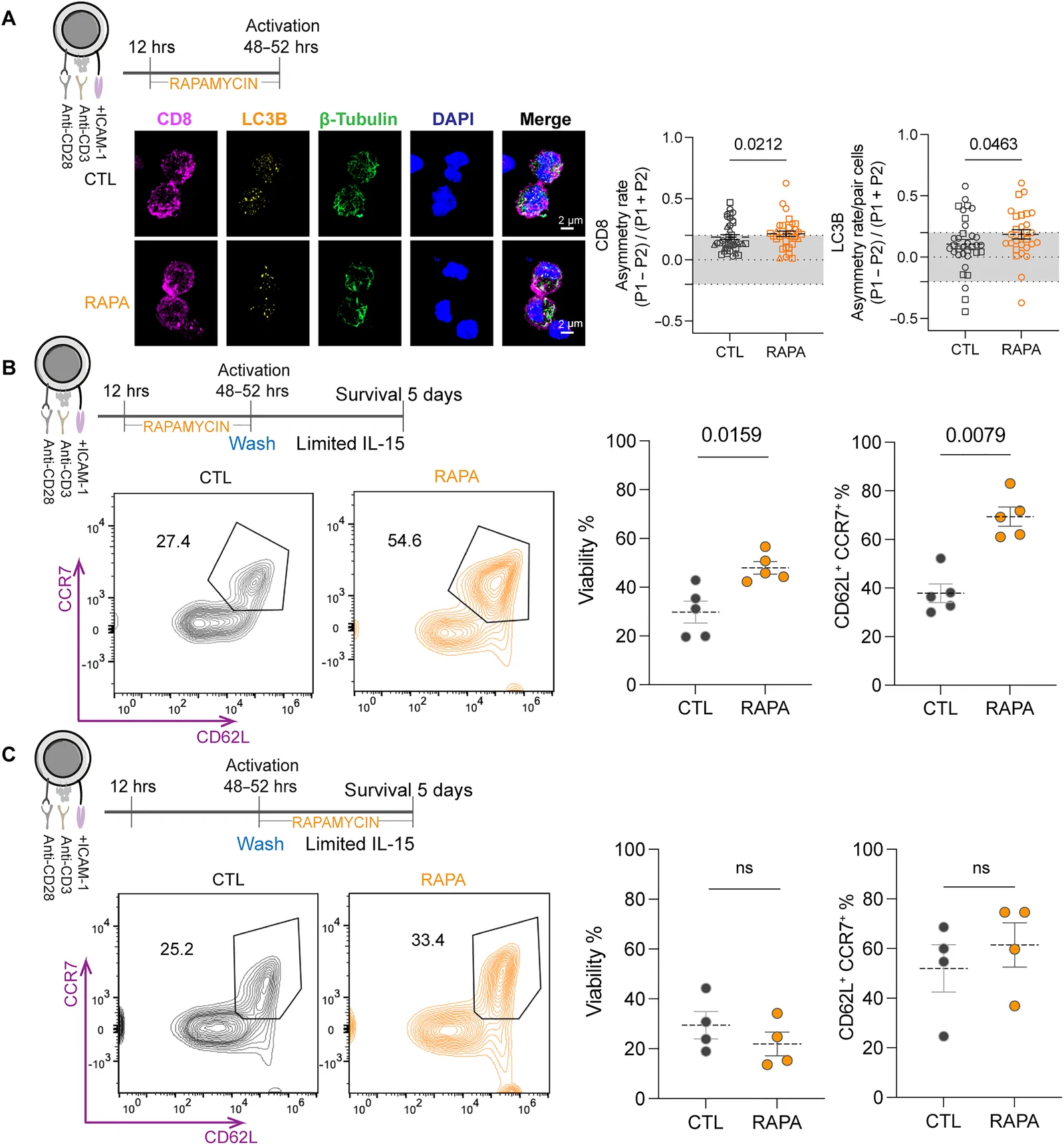
Enhanced ACD boosts T cell memory survival and maintenance. (**A**) CD8^+^ T cells were activated in the presence or absence of rapamycin (20 nM, added 12 hours postactivation) on anti-CD3, anti-CD28, and Fc–ICAM-1–coated plates. Representative confocal images of mitotic CD8^+^ T cells after 48 to 52 hours of activation. CD8 and LC3B asymmetries were calculated between groups with (RAPA) or without (CTL) rapamycin treatment. CTL: *n*D1 = 14, *n*D2 = 15. RAPA: *n*D1 = 10, *n*D2 = 18. (**B**) Isolated CD8^+^ T cells were activated with anti-CD3, anti-CD28 and Fc–ICAM-1 for 48 to 52 hours. Transient rapamycin (20 nM) treatment was added 12 hours after activation. Rapamycin was washed out after 48 to 52 hours, and the cells were cultured for further 5 days in the presence of IL-15 (1 ng/ml). Viability of cells was measured by live/dead staining and compared between CTL and RAPA groups. Representative flow cytometry plots (left) and quantified frequencies of CCR7^+^CD62L^+^ cells (right) compared between CTL and RAPA groups. (**C**) Isolated CD8^+^ T cells were activated with anti-CD3, anti-CD28, and Fc–ICAM-1 for 48 to 52 hours. Cells were harvested and cultured for 5 days in the presence of IL-15 (1 ng/ml) with (RAPA) or without rapamycin (CTL) (20 nM). Viability of cells was measured by live/dead staining and compared between CTL and RAPA groups. Representative flow cytometry plots (left) and quantified frequencies of CCR7^+^CD62L^+^ cells (right) were calculated and compared between groups. Each data point represents one pair of mitosis analyzed (A) or one healthy donor (B and C). Data are presented as means ± SEM. Data points with the same shape represent samples from the same donor. Statistical analysis was performed using Mann-Whitney test. Exact *P* values are depicted in the figure. Data are pooled from two experiments in (A) or representative of one of two experiments in (B) and (C).

## DISCUSSION

The physiological relevance of ACD in T cell immunity has long been debated, primarily because of the lack of tools to track asymmetrically divided daughter cells in vivo and limited evidence from human studies. A recent breakthrough study using the LIPSTIC technique enabled labeling of proximal and distal CAR-T cells, revealing heterogeneity in their fates driven by ACD (*34*). However, direct evidence linking ACD to physiological outcomes in the context of human studies has remained elusive.

Here, we provide the first demonstration that ACD and autophagic machinery are mechanistically linked in shaping human CD8^+^ T cell fate during activation and contributing to vaccine immunogenicity. We demonstrate that ACD in human CD8^+^ T cells leads not only to the unequal partitioning of CD8 but also the autophagic machinery (measured by LC3B), a functionally relevant cell cargo. As CD8 and autophagosomes were frequently coinherited and given the role of autophagy in T cell fate decisions, our results suggest that the asymmetric inheritance of the autophagic machinery contributes to the development of memory T cell phenotypes in vitro and ex vivo. ACD capacity reflected the magnitude of vaccine-induced immune responses, with higher ACD rates associated with better vaccine responsiveness. Enhancing ACD via transient rapamycin treatment significantly improved survival and promoted a memory-like phenotype in CD8^+^ T cell progeny.

Previous studies revealed that ACD involved the asymmetric inheritance of different cellular components, including surface markers (*28*, *34*), cytoplasmic cell cargoes (*24*, *31*, *72*–*74*), transcription factors (*29*, *73*, *75*, *76*), polarity proteins (*77*, *78*), and damaged organelles (*46*). While a definitive answer remains elusive, emerging evidence points to a combination of stochastic, environmental, and cell-intrinsic factors that converge during the first activation and division. The prevailing model posits that ACD in T cells is instructed by the polarization of the immune synapse. Upon encountering an APC, the T cell reorganizes its microtubule-organizing center (MTOC) and secretory machinery toward the synapse (*28*). During the first division, the spindle orientation enables the asymmetric partitioning of fate determinants [e.g., transcription factors (*32*, *79*), metabolic regulators (*73*, *80*), and organelles (*31*, *46*)]. The stability and duration of the T cell–APC interaction, the strength of TCR signaling, and the polarity of the MTOC may vary stochastically or in response to differences in the local microenvironment, even among clonal T cells. Cells with weaker or less stable synapses may fail to establish the robust polarity required for ACD and thus divide symmetrically, for example, as shown in chronic infections, cytokine-induced proliferation (*24*), and in the synapse-deficient model (LFA-1 knockout) (*28*, *32*). However, most of the asymmetric readouts are highly dynamic and the functional link to cell fates in the context of human immunity remains correlative and limited. It is rather challenging to dissect a causal link between ACD and efficient T cell responses, especially in humans. Recent work from our group using a mouse system that allows discrimination between premitotic and postmitotic mitochondria finally demonstrated that asymmetric inheritance of premitotic/older mitochondria predicts T cell fate and is regulated by autophagy in mice (*46*). Extending this concept to human immunity, our data confirm that ACD in human CD8^+^ T cells generates daughter cells with distinct fate trajectories. We demonstrate that the autophagic machinery is segregated during the first mitosis, and this asymmetric inheritance is associated with improved survival and the acquisition of a memory phenotype. A recent report showed that amino acid transport represses autophagy during early murine T cell activation, while autophagy rates are higher in memory cells than in effector cells, supporting the idea that initial autophagic activity does not predict autophagic flux in fate-committed progeny, similar to what we have observed in CD8 hi and CD8 lo first-daughter cells (*44*, *46*).

While the mechanistic basis for autophagy’s role in maintaining memory T cells is still unclear, previous studies have shown that pharmacologic enhancement of autophagy using spermidine improves memory responses in aged mice through inducing TFEB (*39*, *52*). TFEB deficiency has been linked to impaired autophagy during aging (*52*, *81*, *82*), when ACD is also compromised (*33*). We observed an increased expression of TFEB and upregulated autophagic flux in antigen-specific T cells at postvaccination time points. Dynamic changes in GLUT1 expression were also observed across the vaccination time course, suggesting an increased metabolic demand of rapid energy production during T cell activation. Autophagy may contribute to meeting these energetic requirements by sustaining metabolic flexibility, as previously reported in fasting-induced autophagy in aged hematopoietic stem cells to restore glycolysis capacity (*83*). In addition, we detected increased expression of CPT1A during recall responses in antigen-specific T cells, indicating engagement of FAO, a metabolic program known to support mitochondrial function and long-term T cell persistence. Together, these findings suggest that TFEB-driven autophagy coordinates glucose utilization and lipid oxidation to support both acute effector function and memory formation.

Building on earlier mouse studies that linked ACD to long-lived T cell subsets (*32*, *84*), we investigated whether ACD dynamics correlate with vaccine response in humans. We did not observe statistically significant differences in T cell subset composition across different donors and time points before and after vaccination. This is likely due to the small frequency of antigen-specific cells and is reflected in nonsignificant differences in CD8 asymmetry rates when mitoses from cells across different time points were compared. However, we could observe that, in comparison to symmetry controls within the same sample (DAPI control), cells from D0 (prevaccination) showed the most significant differences in ACD rates, reason why we chose this time point to evaluate the ACD potential of each donor within the cohort. To test whether ACD predicts vaccine responsiveness, we stratified donors into high and low responders based on T cell ELISpot data following the first vaccine dose. Strikingly, low responders exhibited significantly reduced ACD. Furthermore, the immune responses assessed in this study, calculated as fold change of vaccine responses between D14 or D7 compared with D0, reflect a combination of both primary and recall responses. However, since individuals participating in the trial have flu-specific Tet^+^ cells at baseline, likely due to annual influenza exposure, we interpret the observed responses as being predominantly driven by memory recall responses. Accordingly, the increased number of IFN-γ–producing cells measured ex vivo in individuals with higher ACD rates should not be interpreted solely as a product of short-lived effector cells originating from a primary response but rather as a functional readout of a robust preexisting memory T cell compartment being reactivated, which will result in a new wave of short-lived effector cells while keeping a memory pool. CD8^+^ T cell subsets can differ in their intrinsic ACD capacity, as suggested by previous studies using murine T cells (*32*). Although subset-specific analysis was not feasible because of clinical sample availability, comparable ACD levels were observed when total and naïve CD8^+^ T cells from healthy donors were compared. Thus, ACD in total CD8^+^ T cells, by reflecting the integrated potential of an individual to have cells undergoing ACD, has been used in this study as a readout of its potential to generate T cell diversity. These findings support the hypothesis that ACD plays a role in maintaining T cell diversity and offer a mechanism where reduced ACD may lead to T cell diversity attrition, limit memory formation and vaccine efficacy in humans.

Although autophagy has long been implicated in T cell survival and metabolic adaptation (*85*), our findings suggest that it is not simply the presence or loss of autophagy but the unequal segregation of autophagic machinery during the first division that may cause early fate bias in CD8^+^ T cells. Selective autophagy, e.g., mitophagy, has been shown to direct early T cell fate decisions. As shown recently by a mouse study from our group, autophagy-dependent mitochondrial inheritance contributes to the divergent cell fates in ACD-derived CD8^+^ T first-daughter cells by regulating metabolic preferences (*46*). Although both lysosomes and autophagosomes participate in selective autophagy, they do not always colocalize, because not all lysosomes are engaged to form autolysosomes. LC3 expression is also not directly proportional to autophagic flux. During T cell activation, for example, LC3 has been shown to regulate internalized LFA-1 trafficking to the contact site and immune synapse formation through an autophagy-independent mechanism (*86*). LFA-1 polarization during ACD has been associated with enhanced memory T cell progeny (*28*), which suggests a possible role for LC3 inheritance in the unequal distribution of signaling components during division, thereby influencing T cell fate independently of canonical autophagy pathways.

This is further strengthened by transient rapamycin treatment administered shortly after T cell activation, which leads to both CD8 and LC3B higher asymmetry rates and increased cell survival. Notably, delayed rapamycin administration (after the first division) had no beneficial effect, demonstrating that the first division is a critical window for fate determination via asymmetric inheritance. Rapamycin has been a promising candidate to enhance immune responses in different immunocompromised scenarios as supported by different clinical trials in the context of vaccination (*69*–*71*, *87*). Although the beneficial effects were observed, the administration timing of rapamycin is diverse, which may lead to different outcomes. It has been reported recently that a 2-week delayed administration of rapamycin following vaccination resulted in superior immune responses compared to immediate postvaccination treatment (*87*). These findings further support the concept that the timing of mTOR inhibition is critical for long-term memory survival. While rapamycin is widely recognized as an autophagy inducer, our findings suggest that its beneficial effects on memory T cells are mediated not by overall increase in autophagy levels but rather perhaps by promoting memory progeny through inducing asymmetric inheritance of autophagic machinery, especially LC3B. Given the central role of mTORC1 in coordinating c-Myc translation (*80*), cytoskeletal organization (*88*), and diffusion barrier formation (*89*), processes closely linked to T cell ACD, rapamycin-mediated inhibition of mTORC1 may represent an upstream regulator of asymmetric fate specification.

Although our data show a significant correlation between ACD and memory responses, the mechanistic link remains to be formally tested. Future studies involving genetic or pharmacological disruption of LC3B localization during ACD will be critical to establish causality. Profiling the specific cargo inherited by daughter cells with high versus low level of autophagy may help clarify how this process shapes functional fate. Since both ACD and autophagy decline with age, these findings could inform strategies to preserve T cell diversity and delay immune aging, potentially affecting systemic aging as well.

In summary, we identify ACD as a fundamental mechanism in human CD8^+^ T cells that facilitates cell fate divergence and supports the generation of memory-prone progeny. These findings offer mechanistic insight into early fate decisions during immune activation and propose ACD as a promising target for enhancing human immune responses including vaccine-induced immunity. Modulating ACD through transient mTOR inhibition may represent an approach to bolster long-term T cell protection, particularly in aged populations.

## MATERIALS AND METHODS

### Human PBMCs

PBMCs samples from the healthy donors (male and female volunteers) were isolated from leukocyte cones ordered from the John Radcliffe Hospital using density gradient centrifugation. Fifteen microliters of cone blood was diluted 1:3 with phosphate-buffered saline (PBS) and layered over Histopaque (Sigma-Aldrich). After centrifugation at 300*g* for 30 min at room temperature without brake, the PBMC layer was collected, washed twice in PBS, and counted using Trypan blue exclusion. PBMCs were then gradient frozen at −80°C or immediately used for experiments.

### FLU005 vaccination trial

The details of vaccination regimens were described previously (*54*). The clinical trial was approved within the UK by the regulatory authority (reference 21,584/0311/001-0001) and the Oxfordshire National Research Ethics Service Committee (OXREC A13/SC/0004). The FLU005 clinical trial (NCT01818362) was a phase 1 trial conducted through the Jenner Institute and Centre for Clinical Vaccinology and Tropical Medicine at the University of Oxford. Written informed consent was obtained from all participants. The studies adhered to the ethical principles outlined in the Declaration of Helsinki (2008) and complied with Good Clinical Practice standards. All investigational vaccines used in the clinical trials were produced by Good Manufacturing Practice. The study enrolled healthy volunteers aged 18 to 50 years (groups 1 to 4, *n* = 46) and those over 50 years of age (group 5 and 6, *n* = 23) with both male and female participants. The trial used a replication-deficient ChAdOx1 vector expressing a fused NP and matrix protein 1 (M1) derived from the influenza A H3N2 strain (A/Panama/2007/99), administered at a dose of 2.5 × 10^10^ viral particles. In addition, an MVA vector encoding NP + M1 was delivered at a dose of 1.5 × 10^8^ plaque-forming units (PFU). This study used PBMCs from groups 1 and 2 and 5 and 6 from this trial, and ELISpot data collected before the start of this study. The details of vaccination groups and regimens are summarized in Table 1.

**Table 1.** FLU005 vaccination groups and regimens.

Group	Age	Number	Prime	Prime dose	Boost	Boost dose	Boost date
1	21–45	12	ChAdOx1 NP + M1	2.5 × 10^10^ v.p.	MVA NP + M1	1.5 × 10^8^ PFU	8 weeks
2	19–39	12	ChAdOx1 NP + M1	2.5 × 10^10^ v.p.	MVA NP + M1	1.5 × 10^8^ PFU	52 weeks
5	52–72	12	ChAdOx1 NP + M1	2.5 × 10^10^ v.p.	-	-	-
6	50–78	12	ChAdOx1 NP + M1	2.5 × 10^10^ v.p.	MVA NP + M1	1.5 × 10^8^ PFU	8 weeks

### T cell isolation, activation, and survival

Total or naïve human CD8^+^ T cells were isolated from PBMCs using magnetic bead–based negative selection following the manufacturer’s instructions (STEMCELL Technologies). Purity was confirmed by flow cytometry (>90%). For T cell proliferation and survival assays, T cells were stained with CellTrace Violet (CTV) at 2.5 μM for 10 min at 37°C and washed with PBS before activation. For activation, 24-well plates were coated with anti-CD3 (OKT3) and anti-CD28 antibodies (5 μg/ml each) with or without human Fc–ICAM-1 (10 mg/ml) overnight and washed with PBS before use. T cells were plated at 1 to 2 × 10^6^ cells/ml and cultured in RPMI 1640 medium supplemented with 10% fetal bovine serum (Sigma-Aldrich) (R10 media), penicillin-streptomycin (Sigma-Aldrich), and hIL-2 (50 U/ml, Peprotech) for 48 to 52 hours. Activated CTV–labeled T cells were harvested and prepared for fluorescence-activated cell sorting (FACS). First division was confirmed by the identification of the first CTV dilution peak. Among first-daughter cells, those exhibiting the 20% highest (CD8 hi) or lowest (CD8 lo) expression were sorted for proliferation and survival assays. For these functional readouts, cells were cultured in medium containing hIL-15 (1 ng/ml) or IL-2 (50 U/ml). Rapamycin treatment was used during activation or survival at 20 nM.

### Tetramer staining

PBMCs from the FLU005 trial were thawed overnight. HLA-A1 or HLA-A2 peptide tetramers were used to stain antigen-specific T cells. Cells were incubated with tetramers in R10 media at 4°C for 30 min, followed by washing and surface staining with fluorochrome-conjugated antibodies. Tetramer-positive cells were analyzed by flow cytometry.

### Surface and intracellular staining for flow cytometry

Cells were incubated with live/dead NIR (Thermo Fisher Scientific) in cold PBS for 10 min at 4°C. Then, the cells were washed using FACS buffer [PBS with 2% bovine serum albumin (BSA) and 2 mM EDTA (Thermo Fisher Scientific)] and centrifuged at 500*g* for 5 min. Cells were incubated with fluorophore-conjugated antibody mix targeting surface markers for 30 min at 4°C. PBMCs were stained with antibodies against CD3, CD4, CD8, CD19, CD14, CD16, CD56, HLA-DR, CD11c, CD45RA, CD45RO, CCR7, CD62L, and CD57. Purified and activated CD8^+^ T cells were stained with antibodies against CD8, CD25, CD44, CD62L, CD45RA, CD45RO, CD57, and CD27. For intracellular staining, the cells were fixed and permeabilized using a commercial fixation/permeabilization kit following manufacturer’s instructions (Waters Biosciences, former BD Biosciences) and stained with antibodies against intracellular proteins (TFEB, TOM20, CPT1A, IFN-γ, and IL-2). Samples were acquired on a flow cytometer (BD LSR-II, Fortessa X-20, Cytek Aurora) and analyzed with FlowJo software. Details of reagents, softwares, and equipments used in this study are listed in Table 2.

**Table 2.** Reagents, software, and equipment.

Reagent	Supplier	Catalog number	Dilution	Clone
CD8	BioLegend	344726	1:200	SK1
DAPI	Thermo Fisher Scientific	62248	1:2000	-
α-Tubulin	Sigma-Aldrich	T6199	1:500	DM1A
β-Tubulin	Sigma-Aldrich	T5293	1:500	2-28-33
LAMP-1	Abcam	ab25630	1:500	H4A3
LC3B	CST	83506	1:1000	E5Q2K
TFEB	CST	4240	1:1000	-
GLUT1	Abcam	ab210438	1:100	EPR3915
TOM20	Abcam	ab209606	1:100	EPR15581-54
p62	CST	5114 T	1:1000	-
Goat-anti-rabbit AF568 immunoglobulin G (IgG)	Invitrogen	A-11011	1:1000	-
Donkey-anti-mouse AF488 IgG	Invitrogen	A-21202	1:1000	-
Live/Dead Blue	Invitrogen	L34976	1:1000	-
Live/Dead NIR	Invitrogen	L34962	1:1000	-
Histopaque	Sigma-Aldrich	10771	-	-
Red blood cell lysis buffer	Thermo Fisher Scientific	50-112-9751	-	-
Fetal bovine serum	Sigma-Aldrich	F9665	-	-
DMSO	Sigma-Aldrich	D2650	-	-
Easysep human CD8^+^ T cell isolation kit	Stem Cell Technologies	17953	-	-
Easysep human naïve CD8^+^ T cell isolation kit	Stem Cell Technologies	17968	-	-
Easysep human memory CD8^+^ T cell enrichment kit	Stem Cell Technologies	19159	-	-
Autophagy LC3 antibody-based assay Kit	Cytek Biosciences	FCCH100171	-	-
Click-iT OPP kit	Thermo Fisher Scientific	c10457	-	-
RPMI 1640	Sigma-Aldrich	R8758	-	-
Hepes buffer	Thermo Fisher Scientific	15630–056	-	-
Sodium pyruvate	Sigma-Aldrich	S8636	-	-
B-mercaptoethonal	Thermo Fisher Scientific	31350–010	-	-
Nonessential amino acid	Thermo Fisher Scientific	11140035	-	-
Penicillin streptomycin	Sigma-Aldrich	P0781	-	-
4% PFA	Thermo Fisher Scientific	R37602	-	-
Human IL-2	Peprotech	200–02	-	-
Human IL-7	Peprotech	200–07	-	-
Human IL-15	Peprotech	200–15	-	-
Anti-human CD3	BioLegend	300331	-	-
Anti-human CD28	BioLegend	302934	-	-
Human Fc–ICAM-1	R&D	720-IC-200	-	-
Poly-l-lysine	Sigma-Aldrich	P8920	-	-
Rapamycin	Cayman Chemical	53123–88-9	-	-
Bafilomycin a1	Sigma-Aldrich	SML-1661	-	-
Chloroquine	Sigma-Aldrich	C6628- 25G		
Flow cytometry antibody	Supplier	Fluorochrome	Dilution	Catalog number
CD8	BioLegend	Alexa Fluor 647	1:200	344726
CD4	BioLegend	Alexa Fluor 700	1:200	103025
CD25	BioLegend	BV711	1:200	356138
CD27	BioLegend	BV650	1:200	302827
CD62L	BioLegend	BV510	1:200	304743
CD45RA	BioLegend	PeCy7	1:200	304125
CD45RO	BioLegend	BV510	1:200	304245
Cell Trace Violet	Invitrogen	-	1:1000	C34557
CD57	BioLegend	Alexa Fluor 647	1:200	359614
CD3	BD Bioscence	BUV395	1:400	563548
TCF1	BD Bioscence	PE	1:400	564217
CD4	BioLegend	APC	1:200	317416
CD19	BioLegend	PeCy5	1:200	302210
CD16	BioLegend	PE	1:200	980102
CD11c	BD Bioscence	BUV496	1:200	741139
HLA-DR	BioLegend	APC/Cy7	1:200	307617
CD56	BioLegend	BV650	1:200	362531
CD14	BioLegend	BV711	1:200	301838
CD28	BD Bioscence	BUV661	1:200	741635
Goat-anti-rabbit IgG	Thermo Fisher Scientific	Alexa Fluor 532	1:100	A11009
CCR7	BioLegend	PerCP/Cy5.5	1:200	353219
CD27	Thermo Fisher Scientific	efluoro 710	1:200	46–0279-42
Software	Version			
FlowJo	10.10.0			
ImageJ	1.54 m			
GraphPad Prism	10.0.3 (217)			
Instrument				
Zeiss 980 Airyscan				
BD Fortessa X-20				
BD LSR-II				
Cytek Aurora	5 L UV-V-B-YG-R			
BD Aria-III				

### T cell IFN-γ ELISpot

Ex vivo T cell ELISpot assays were conducted using freshly isolated PBMCs to assess immune responses to the NP + M1 vaccine antigen at each study time point (*90*). To evaluate the breadth of the NP + M1–specific T cell response, eight peptide pools were used. Each pool consisted of 10 overlapping 15 to 20–amino acid peptides, with a 10-amino acid overlap between consecutive peptides, collectively spanning the entire NP + M1 insert as previously described (*54*). T cell ELISpot data were acquired from the Oxford Vaccine Group.

### Autophagy flux assay for flow cytometry

Autophagy flux was assessed by detecting LC3-II accumulation using BafA (10 nM, 2 hours), as described before (*45*). Briefly, the cells were treated with or without BafA for 2 hours. Afterward, the cells were stained with surface markers and washed with LC3 assay buffer (Cytek) in 96-well U bottom plates. One hundred microliters of 1× Reagent B, as provided in the kit, was added to each well, followed by immediate centrifugation. The cells were then incubated with anti-LC3 (fluorescein isothiocyanate, FITC) antibody at 1:20 in assay buffer (30 to 50 μl per well) at room temperature for 30 min and washed once with 150 μl of assay buffer. Stained cells were fixed with 2% paraformaldehyde (PFA) before FACS analysis. Autophagic flux was calculated as LC3-II mean fluorescence intensity of (BafA-vehicle)/ vehicle.

### Click-iT OPP assay (translation activity)

T cell translation activity was measured using the Click-iT OPP assay (Thermo Fisher Scientific) according to the manufacturer’s instructions. Briefly, activated CD8^+^ T cells were plated in a U-bottom 96-well plate and incubated with Click-iT OPP for 30 min in R10 medium in the presence or absence of cycloheximide, a translation inhibitor. Cells were then washed and stained for surface markers for downstream flow cytometry analysis as described above. After being washed with PBS, cells were fixed with 4% PFA (in PBS) for 15 min at room temperature, followed by permeabilization with 0.5% Triton X-100 (in PBS) for 15 min. Fresh reaction cocktail was prepared according to the manufacturer’s instructions. Cells were incubated with the reaction cocktail for 30 min at room temperature and protected from light. After incubation, cells were washed with Click-iT Reaction Rinse Buffer and resuspended in FACS buffer before flow cytometry analysis. Detailed information about the reagents and buffers can be found in the list of reagents/buffers (Table 2). The antibodies used can be found in the list of antibodies for OPP assay (Table 2).

### Immunofluorescence

Human CD8^+^ T lymphocytes were washed with PBS and added on coverslips treated with poly-l-lysine, followed by 30 min incubation at 37°C. Cells were then fixed with 2% PFA (Sigma-Aldrich) for 10 min, permeabilized with 0.15% Triton X-100 (Sigma-Aldrich) for 10 min, and blocked in PBS containing 2% BSA and 0.01% Tween 20 for 1 hour at room temperature. Primary antibodies [anti-human CD8 Alexa Fluor 647 (BioLegend, clone: SK1), anti–β-tubulin (Sigma-Aldrich), anti-LC3B (Cell Signaling Technology), anti-LAMP1 (Abcam), and anti-TFEB (Cell Signaling Technology)] were diluted in PBS containing 2% BSA and incubated overnight at 4°C, followed by 1 hour of incubation at room temperature with secondary antibodies [(donkey-anti-mouse-Alexa Fluor 488 (Thermo Fisher Scientific) and goat-anti-rabbit–Alexa Fluor 568 (Thermo Fisher Scientific)]. Nuclei were stained with DAPI (Thermo Fisher Scientific). Mitotic cells were identified and acquired as previously described (*32*). Briefly, mitotic cells were identified by condensed chromosome morphology, the presence of two microtubule organizing centers, and a clear tubulin bridge between two daughter cells. Images were acquired using a confocal microscope (Zeiss-980 Airyscan) and analyzed with ImageJ. Asymmetry rates were calculated on the basis of the quantification of asymmetry markers (volume and fluorescence intensity) in either hemisphere of the mitotic pair. P1 was defined as the pole containing higher amounts of CD8, and P2 was defined as the opposite pole. Mitoses were considered asymmetric when CD8 enrichment was 1.5-fold greater in one daughter cell in comparison with the other. 1.5-fold is when (P1 − P2) / (P1 + P2) = 0.2.

### RNA sequencing

Total RNA of CD8 hi and CD8 lo cells was extracted using the QIAGEN RNA isolation micro kit. RNA integrity was confirmed using an Agilent Bioanalyzer. To generate PolyA libraries, cDNA was end repaired, A tailed, and adapter ligated. Libraries were then size selected, multiplexed, quality controlled, and sequenced using a NovaSeq 6000. Quality control of raw reads was performed with a pipeline readqc.py (https://github.com/cgat-developers/cgat-flow). The resulting reads were aligned to the GRCh38 (hg38) reference genome using the pseudoalignment method Salmon (*91*). Quantified transcript counts were then imported into DESeq2 (v1.38.3) for differential expression analysis, following the standard workflow of size factor estimation, dispersion estimation, and fitting of a negative binomial generalized linear mod (*92*). To correct for interdonor variability, batch effects were removed before testing using the limma package (v3.54.1), which applies linear models to adjust for unwanted variation (*93*). Genes with an adjusted *P* value <0.05 after Benjamini-Hochberg correction were considered significantly differentially expressed. Functional interpretation was performed by Gene Set Enrichment Analysis using clusterProfiler (v4.6.0), which integrates curated gene sets to reveal enriched biological pathways (*94*). Standard pipeline was executed according to the DESeq2 tutorial. Genes linked to T cell differentiation were selected on the basis of previous studies (*10*, *95*, *96*) and were differentially expressed in our dataset.

### Statistical analysis

Data were analyzed using GraphPad Prism. To test whether data point values were distributed in a Gaussian distribution, we performed D’Agostino-Pearson and Kolmogorov-Smirnov normality test. For statistical analysis, two-tailed Student’s *t* test or nonparametric analysis Mann-Whitney *U* tests were used. For multiple group comparisons, we performed one-way analysis of variance (ANOVA) with Welch test and Brown-Forsythe test or nonparametric analysis Kruskal-Wallis. For multieffects analysis, we performed two-way ANOVA. Differences were regarded as significant when *P* < 0.05.
