# Supplementary material for: Asymmetric cell division is predictive of human T cell vaccine responses
Source: Sci Adv. 2026 Nov 11;12(46):eaef1608. doi: 10.1126/sciadv.aef1608 (PMC13753999; doi:10.1126/sciadv.aef1608)
Supplement: Supplementary file 1 [file sciadv.aef1608_sm.pdf]

Science Advances
AAAS

Supplementary Materials for

Asymmetric cell division is predictive of human T cell vaccine responses

Lin Luo *et al.*

Corresponding author: Teresa Lambe, teresa.lambe@paediatrics.ox.ac.uk;
Anna Katharina Simon, katja.simon@mdc-berlin.de; Mariana Borsa, mariana.borsa@unibas.ch

Sci. Adv. **12**, eaef1608 (2026)
DOI: 10.1126/sciadv.aef1608

The PDF file includes:

Figs. S1 to S6
Legend for data S1

Other Supplementary Material for this manuscript includes the following:

Data S1

Figure S1. Validation of first division, OPP assay and phenotypes of memory differentiation conditions. (A) Isolated total $CD8^+$ T cells were cultured in R10 medium with anti-CD3, anti-CD28, and Fc-ICAM-1 stimulation for 36 hrs (black solid line) or 52 hrs (grey). Cell Trace Violet (CTV) dilution histograms were analyzed for the first division by flow cytometry. (B) Validation of transcription factor staining for the memory precursor phenotype under fluorescence-minus-one (FMO) staining control, -ICAM-1 and +ICAM-1 conditions. (C-E) Comparisons of double-positive populations ($CD62L^+CCR7^+$, $CD62L^+TCF\text{-}1^+$, or $CD62L^+FOXO1^+$) representing memory fate between -ICAM-1 and +ICAM-1 conditions. (F) Validation of OPP assay. OPP intensity was measured by flow cytometry in FMO, in presence of translation inhibitor cycloheximide (CHX), and in the control group (left). (G) Histograms of OPP intensity by flow cytometry in CD8 hi (green) and CD8 lo (purple) cells. (H) PCA of differentially expressed genes (DEGs) between CD8 hi and CD8 lo cells. Each data point represents one healthy donor. Statistical analysis was performed using a paired t-test (C-E). Exact P values are depicted in the figure.

Figure S1

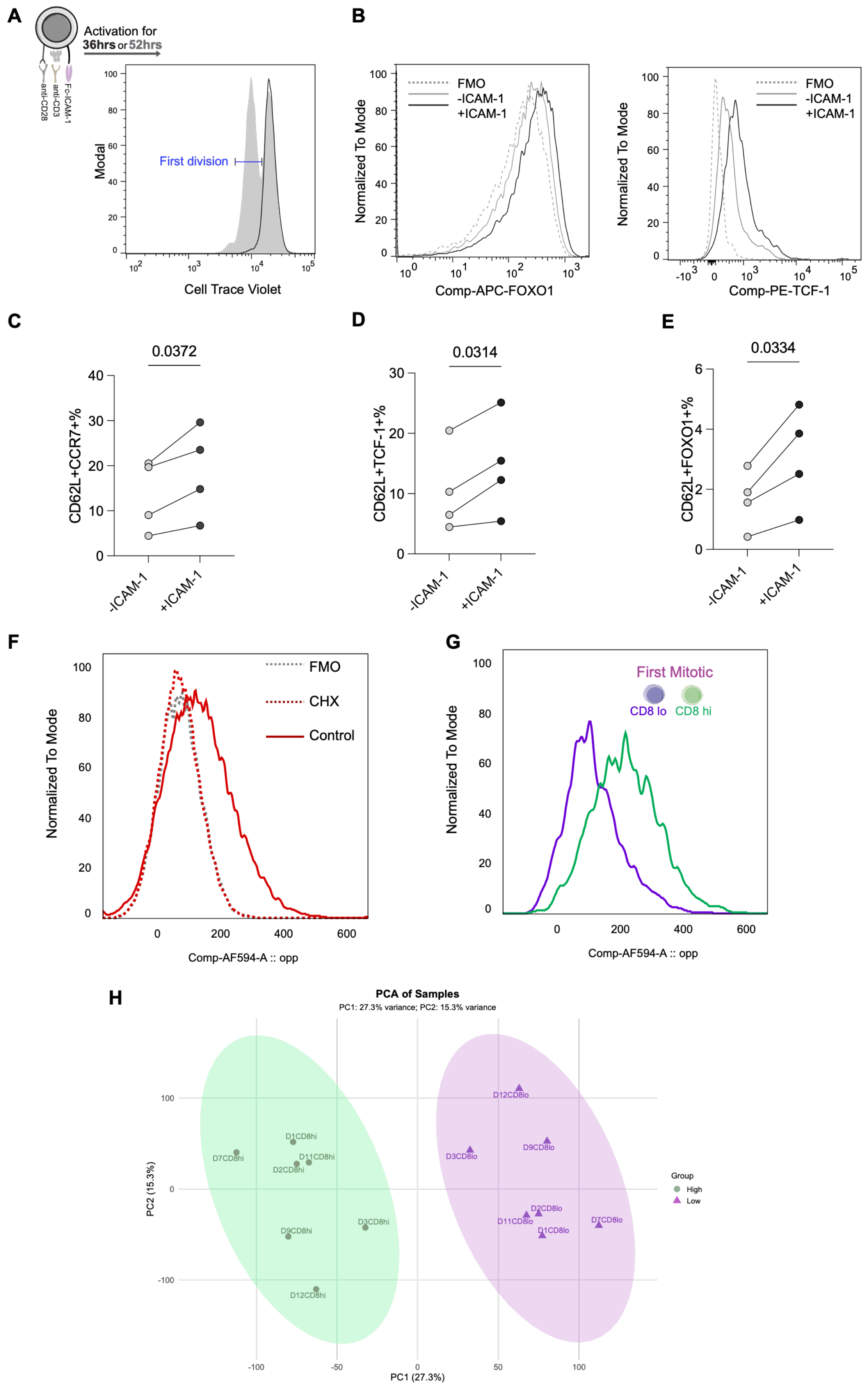

A
Activation for
36hrs or 52hrs
anti-CD28
anti-CD3
Fc-ICAM-1
Modal
First division
Cell Trace Violet
B
FMO
-ICAM-1
+ICAM-1
Normalized To Mode
Comp-APC-FOXO1
Comp-PE-TCF-1
C
0.0372
CD62L+CCR7+%
-ICAM-1
+ICAM-1
D
0.0314
CD62L+TCF-1+%
E
0.0334
CD62L+FOXO1+%
F
FMO
CHX
Control
Comp-AF594-A :: opp
G
First Mitotic
CD8 lo
CD8 hi
H
PCA of Samples
PC1: 27.3% variance; PC2: 15.3% variance
PC1 (27.3%)
PC2 (15.3%)
Group
High
Low

Figure S2. Co-inheritance of CD8 and autophagic machineries. (A) Correlation analysis of LAMP-1 and CD8 asymmetries. (B) Correlation analysis of LC3B and CD8 asymmetries. (C) Bafilomycin A1 (BafA) was added at the last 2 hrs of activation during ACD before harvesting for microscopy. LC3B asymmetry was not affected by BafA treatment. (D) Graphical summary of autophagic flux assay: BafA treatment inhibits autophagic flux and results in higher MFI of LC3-II (Upper panel). Cell viability of $CD8^{+}$ T cells under control (CTL) and BafA conditions following the autophagic flux staining (Lower panel). (E) LC3-II MFI for CD8 hi and lo cells 3 days post-first division. (F) LC3-II MFI for CD8 hi and lo cells 7 days post-first division. (G) Heatmap showing expression ratios of autophagy-associated genes among donors (fold change CD8 hi vs CD8 lo). Each data point represents one pair of mitosis analyzed (A, B, C) or one healthy donor (E, F). Data are presented as mean±SEM. Statistical analysis was performed using Mann-Whitney test (C), Spearman correlation co-efficient test (A, B), and Kruskal-Wallis with Dunn's correction (E, F, G). Exact P values are depicted in the figure. Data are pooled from 2 experiments in (A, B, C) and representative in (E, F) of 1 experiment out of 2.

Figure S2

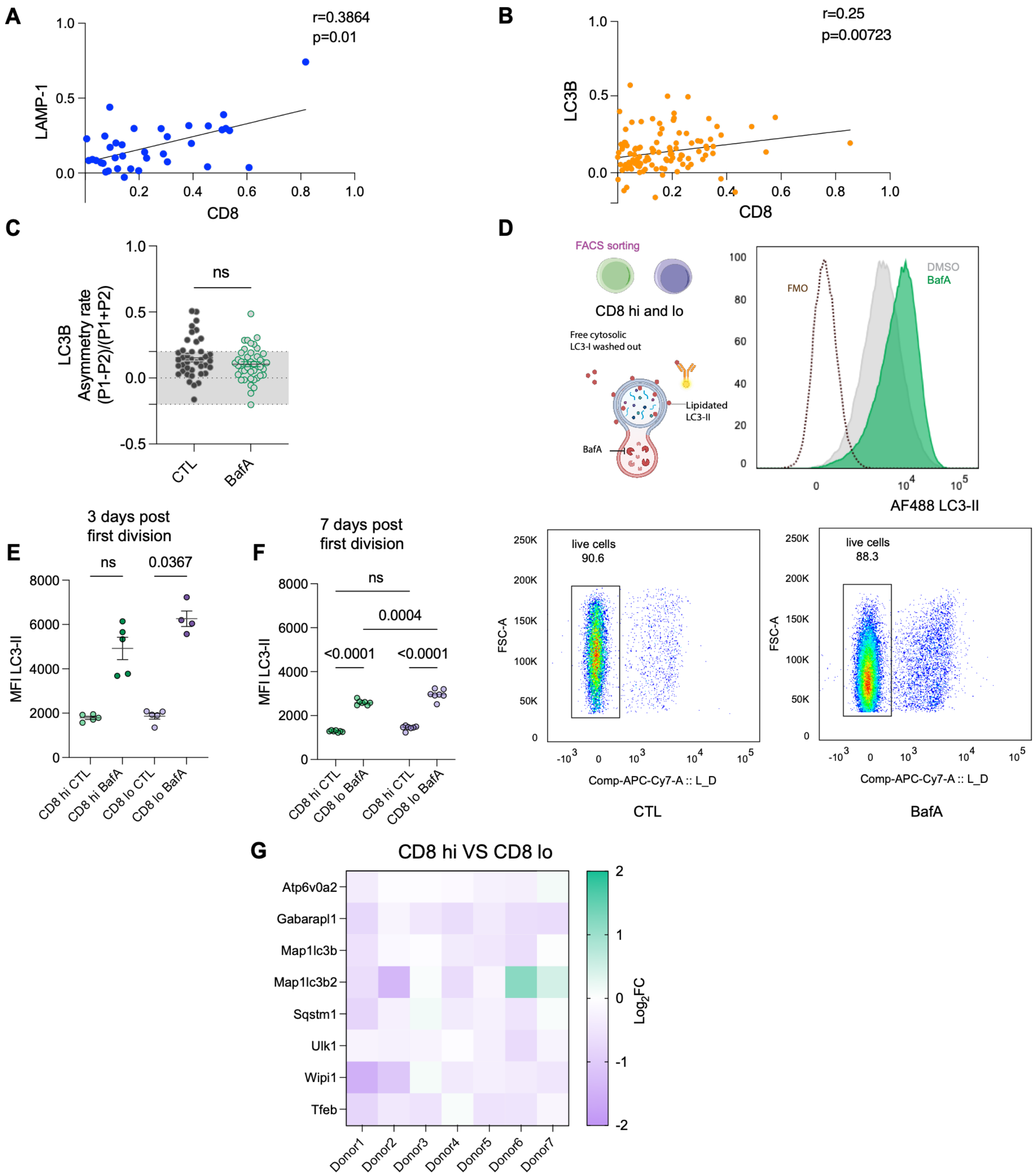
A
r=0.3864
p=0.01
LAMP-1
CD8
B
r=0.25
p=0.00723
LC3B
CD8
C
LC3B
Asymmetry rate
(P1-P2)/(P1+P2)
ns
CTL
BafA
D
FACS sorting
CD8 hi and lo
Free cytosolic
LC3-I washed out
Lipidated
LC3-II
BafA
FMO
DMSO
BafA
AF488 LC3-II
live cells
90.6
live cells
88.3
FSC-A
Comp-APC-Cy7-A :: L_D
CTL
BafA
E
3 days post
first division
ns
0.0367
MFI LC3-II
CD8 hi CTL
CD8 hi BafA
CD8 lo CTL
CD8 lo BafA
F
7 days post
first division
ns
0.0004
<0.0001
<0.0001
MFI LC3-II
CD8 hi CTL
CD8 lo BafA
CD8 hi CTL
CD8 lo BafA
G
CD8 hi VS CD8 lo
Atp6v0a2
Gabarapl1
Map1lc3b
Map1lc3b2
Sqstm1
Ulk1
Wipi1
Tfeb
Donor1
Donor2
Donor3
Donor4
Donor5
Donor6
Donor7
Log2FC

Figure S3. Chloroquine treatment impairs T cell survival and disrupts memory-associated but not effector-associated phenotypes in first daughter cells. (A) Experimental setup of Chloroquine (CQ) treatment in first-division daughter cells. Briefly, naïve T cells were isolated and activated for 52 hrs and sorted for CD8 hi and CD8 lo cells, followed by 24 hrs rest in survival medium. Cells were then cultured for 3 days in absence of any TCR stimulation and in presence or absence of CQ before examining their viability and phenotypes. (B) Cell viability analysis for sorted $CD8^+$ T cells after 3 days *in vitro* survival with or without CQ treatment. (C-F) Phenotype analysis of surviving cells in (B). Data are presented as mean±SEM. Each data point represents one healthy donor. Statistical analysis was performed using Student's t-test (B–F). Exact P values are depicted in the figure. Data are representative in of 1 experiment.

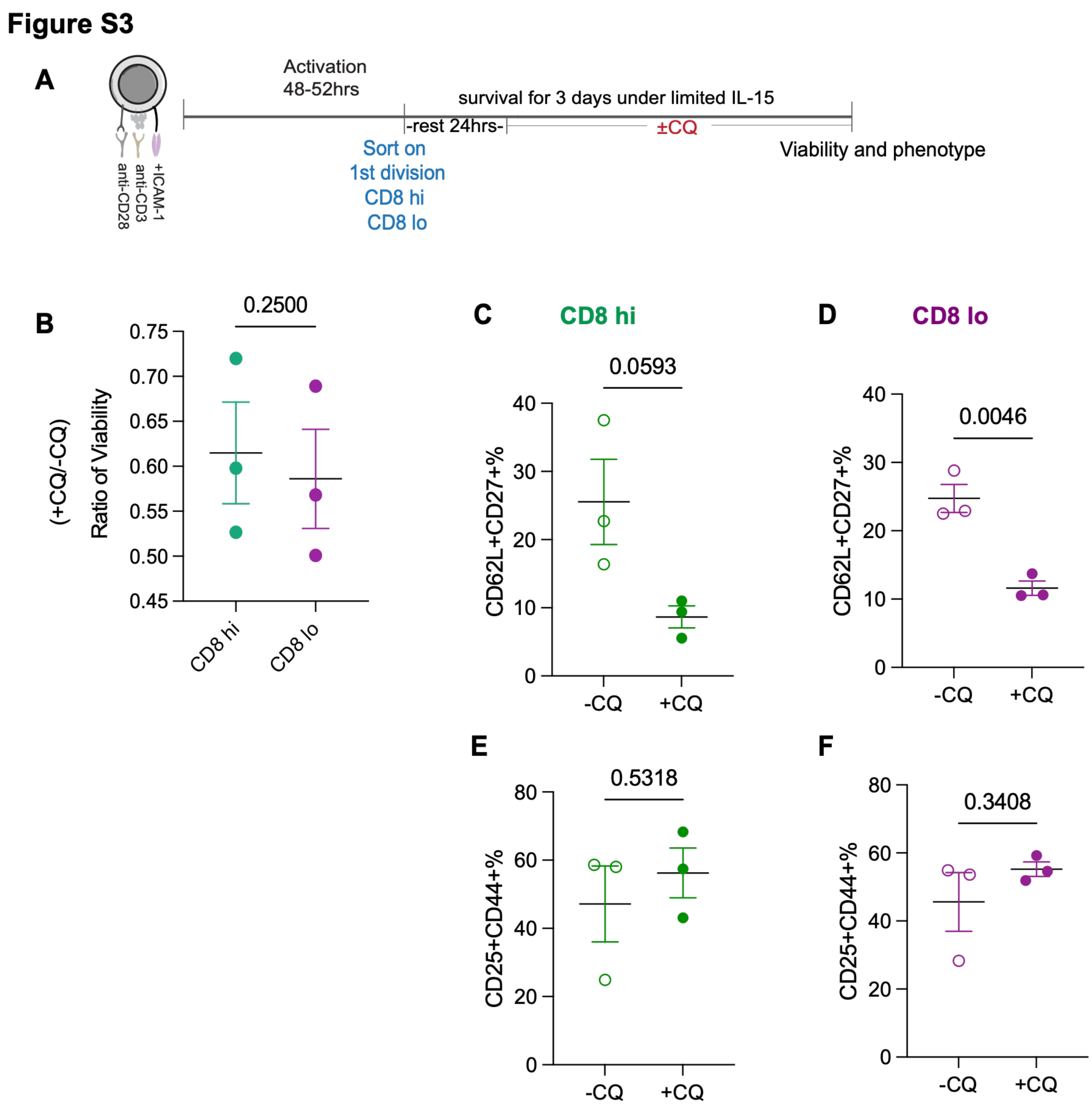

Figure S4. Gating strategy and phenotyping of Tet$^+$ CD8$^+$ T cells. (A) Gating strategy of CD8$^+$ T cell subsets from healthy PBMCs. (B) Gating strategy of tetramer-positive (Tet$^+$) population within CD8$^+$ T cells found in PBMCs from FLU005 vaccinees, representing antigen-specific T cells. (C) Percentages of CD45RA$^+$ and CD45RO$^+$ in total Tet$^+$ cells at D63. (D) Comparison of autophagic flux among CD45RA$^+$, CD45RO$^+$ and total Tet$^+$ cells. Data are presented as mean±SEM. Each data point represents one vaccinee. Statistical analysis was performed using Student's t-test (C) or Kruskal-Wallis test (D). Exact P values are depicted in the figure. Data are representative in of one experiment.

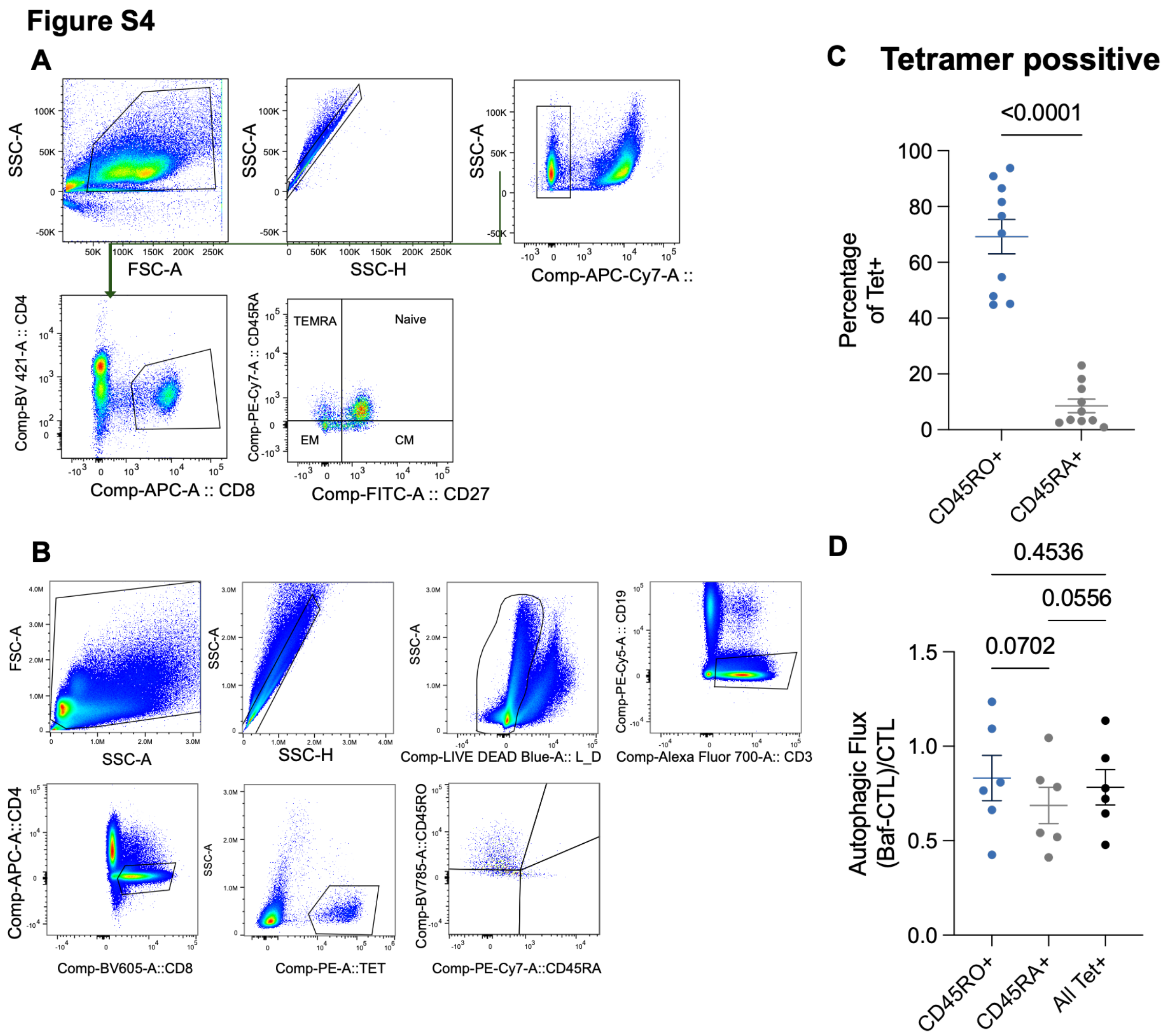

Figure S5. T cell subset dynamics and vaccine response correlates in FLU005 vaccinees (related to Figure 5). (A) Analysis of T cell subsets in total $CD8^+$ T cells from FLU005 trial. $CD8^+$ T cell subsets were analyzed from *ex vivo* PBMCs at different time points (D0, D14, D182). Naïve, central memory (CM), effector memory (EM), and terminally differentiated effector memory (TEMRA) were identified as $CD8^+CD45RA^+CCR7^+$, $CD8^+CD45RO^+CD27^+$, $CD8^+CD45RO^+CD27^-$, and $CD8^+CD45RA^+CD27^-$, respectively. The percentage of T cell subsets to total $CD8^+$ T cells was calculated. (B) Percentage of tetramer-positive cells within total $CD8^+$ T cells was calculated at different time points from groups that received single (left) or double (right) doses of influenza vaccination. Each data point represents one healthy donor. (C) Comparisons of CD8 asymmetry between total and naïve T cells from four healthy donors. (D) Definition of high (black dots) and low responders (orange dots) based on the median fold change of T cell ELISpot D7 or D14 vs D0. (E) Pearson correlation of individuals' vaccine response (fold change, D7 or D14 vs D0) and ACD%. High (black dots) and low responders (orange dots) are depicted in different colors. (F) Percentage of ACD in total $CD8^+$ T cells was calculated and compared between young (Y) and older (O) donors. (G) Comparison of vaccine response fold change (FC, D7/D0) after the first dose between young and older donors. (H) Comparisons of autophagic flux between high HR and LR groups in total $CD8^+$ T cells from FLU005 vaccinees at D56. Each data point represents one healthy donor. Data are presented as mean±SEM. Statistical analysis was performed using Two-way ANOVA with Šídák's multiple-comparison correction (A), Kruskal-Wallis test (B, C), Mann-Whitney test (F, G, H) and Pearson correlation co-efficienct test (E). Exact P values are depicted in the figure.

Figure S5

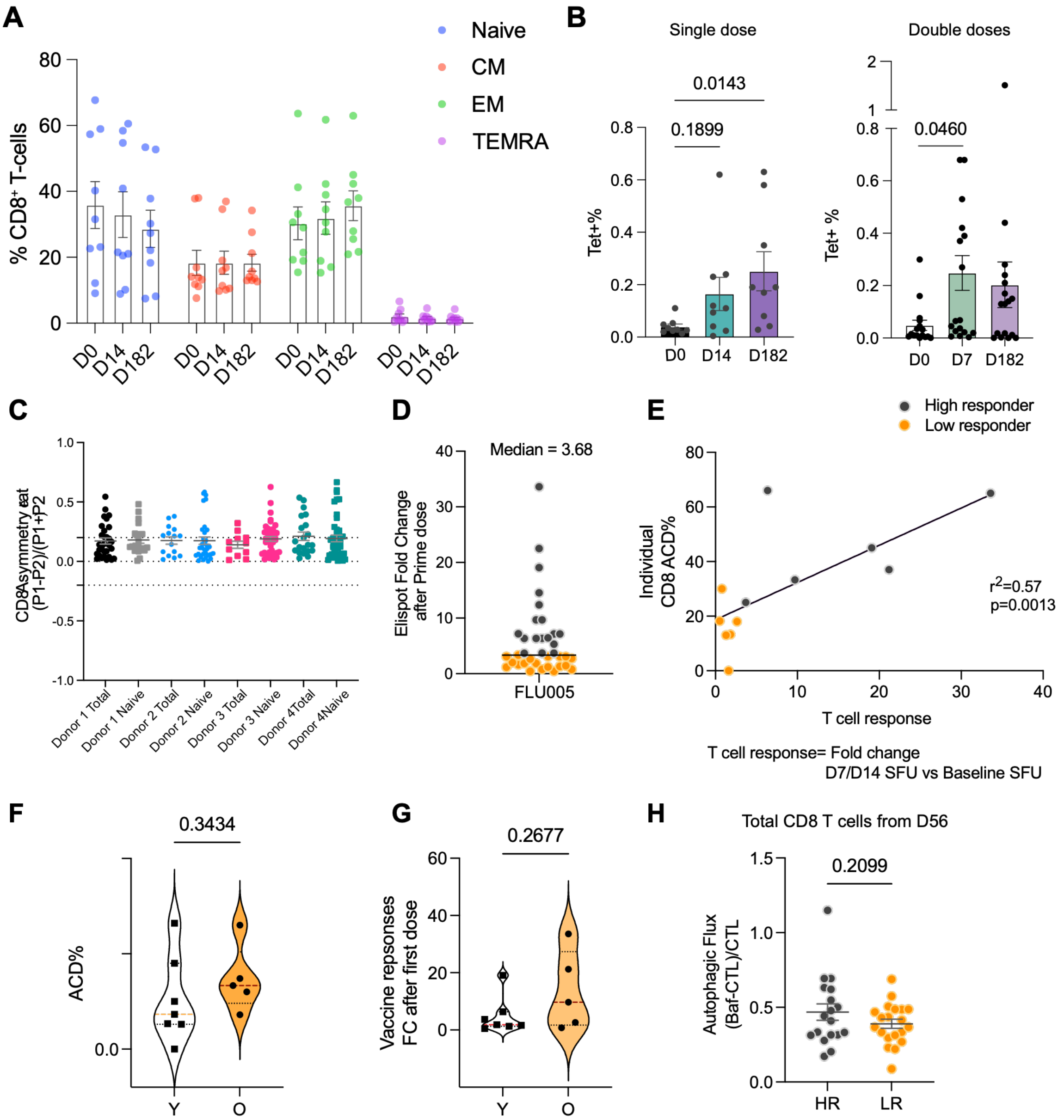
A
Naive
CM
EM
TEMRA
% CD8+ T-cells
D0
D14
D182
B
Single dose
Double doses
0.0143
0.1899
0.0460
Tet+%
Tet+ %
D0
D14
D182
D7
C
CD8 Asymmetry rat
(P1-P2)/(P1+P2
Donor 1 Total
Donor 1 Naive
Donor 2 Total
Donor 2 Naive
Donor 3 Total
Donor 3 Naive
Donor 4Total
Donor 4Naive
D
Median = 3.68
Elispot Fold Change after Prime dose
FLU005
E
High responder
Low responder
Individual CD8 ACD%
r2=0.57
p=0.0013
T cell response
T cell response= Fold change D7/D14 SFU vs Baseline SFU
F
0.3434
ACD%
Y
O
G
0.2677
Vaccine repsonses FC after first dose
H
Total CD8 T cells from D56
0.2099
Autophagic Flux (Baf-CTL)/CTL
HR
LR

Figure S6. Transient rapamycin treatment does not induce autophagy or memory-like cell proliferation. (A) Experimental design of *in vitro* survival assay, when $CD8^+$ T cells were treated with or without rapamycin during activation. (B-C) MFI of CTV was quantified in different $CD8^+$ T cell subsets exposed to rapamycin treatment or not (with: RAPA, without: CTL). (D-E) Comparison of autophagic flux in total $CD8^+$ T cells and memory-like ($CCR7^+CD62L^+$) $CD8^+$ T cell populations treated or not with rapamycin after 5 days in culture (survival assay described in A). Each data point represents one healthy donor. (F) Experimental design of *in vitro* survival assay, when $CD8^+$ T cells were treated with rapamycin alone or combined with chloroquine (CQ) during activation. (G-H) Cell viability was assessed after 5 days of survival with low (G, 10μM) and high CQ (H, 10mM) under the conditions in (F). Data are presented as mean±SEM. Statistical analysis was performed using Mann-Whitney test (D, E) and Kruskal-Wallis test (G, H), and two-way ANOVA test (C). Exact P values are depicted in the figure. Representative of one experiment out of two.

Figure S6

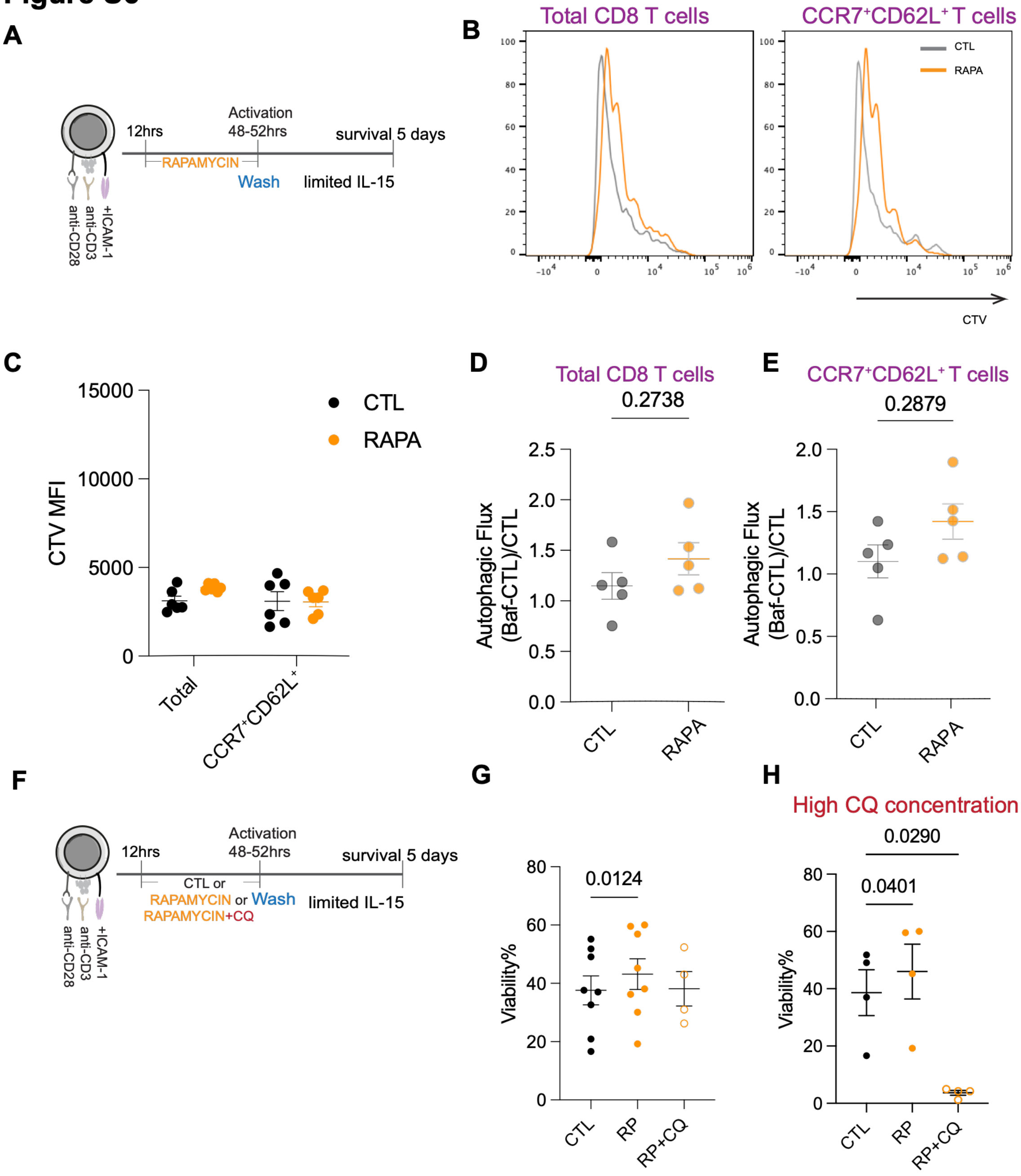
A
Activation
48-52hrs
12hrs
survival 5 days
RAPAMYCIN
Wash
limited IL-15
anti-CD28
anti-CD3
+ICAM-1
B
Total CD8 T cells
CCR7+CD62L+ T cells
CTL
RAPA
CTV
C
CTV MFI
CTL
RAPA
Total
CCR7+CD62L+
D
Total CD8 T cells
0.2738
Autophagic Flux (Baf-CTL)/CTL
CTL
RAPA
E
CCR7+CD62L+ T cells
0.2879
Autophagic Flux (Baf-CTL)/CTL
CTL
RAPA
F
Activation
48-52hrs
12hrs
survival 5 days
CTL or
RAPAMYCIN or Wash
RAPAMYCIN+CQ
limited IL-15
anti-CD28
anti-CD3
+ICAM-1
G
0.0124
Viability%
CTL
RP
RP+CQ
H
High CQ concentration
0.0290
0.0401
Viability%
CTL
RP
RP+CQ

Supplementary Materials:

Raw data of the main figures and supplementary figures could be found in supplementary_materials.xlsx
